# Myofiber directs macrophages IL-10-Vav1-Rac1 efferocytosis pathway in inflamed muscle following CTX myoinjury by activating the intrinsic TGF-β signaling

**DOI:** 10.1186/s12964-023-01163-8

**Published:** 2023-07-04

**Authors:** Zhaohong Liao, Haiqiang Lan, Xiaoting Jian, Jingwen Huang, Han Wang, Jijie Hu, Hua Liao

**Affiliations:** 1 Guangdong Provincial Key Laboratory of Construction and Detection in Tissue Engineering; Department of Anatomy, School of Basic Medical Sciences, Southern Medical University, Guangzhou, 510515 China; 2 Department of Laboratory Medicine, School of Medicine, Foshan University, Foshan, 528000 China; 3 Department of Orthopedics and Traumatology, Nanfang Hospital, Southern Medical University, Guangzhou, 510515 China

**Keywords:** TGF-β signaling, Macrophages efferocytosis, Myoinjury, IL-10

## Abstract

**Background:**

To explore the role of skeletal muscle specific TGF-β signaling on macrophages efferocytosis in inflamed muscle caused by Cardiotoxin (CTX) injection.

**Methods:**

CTX myoinjury was manipulated in TGF-βr2^flox/flox^ (control) mice or transgenic mice with TGF-β receptor 2 (TGF-βr2) being specifically deleted in skeletal muscle (SM TGF-βr2^−/−^). Gene levels of TGF-β signal molecules, special inflammatory mediators in damaged muscle or in cultured and differentiated myogenic precursor cells (MPC-myotubes) were monitored by transcriptome microarray or qRT-PCR. TGF-β pathway molecules, myokines and embryonic myosin heavy chain in regenerating myofibers, the phenotype and efferocytosis of macrophages were evaluated by immunofluorescence, immunoblotting, Luminex, or FACS analysis. In vitro apoptotic cells were prepared by UV-irradiation.

**Results:**

In control mice, TGF-β-Smad2/3 signaling were significantly up-regulated in regenerating centronuclear myofibers after CTX-myoinjury. More severe muscle inflammation was caused by the deficiency of muscle TGF-β signaling, with the increased number of M1, but the decreased number of M2 macrophages. Notably, the deficiency of TGF-β signaling in myofibers dramatically affected on the ability of macrophages to conduct efferocytosis, marked by the decreased number of Annexin-V^−^F4/80^+^Tunel^+^ macrophages in inflamed muscle, and the impaired uptake of macrophages to PKH67^+^ apoptotic cells transferred into damaged muscle. Further, our study suggested that, the intrinsic TGF-β signaling directed IL-10-Vav1-Rac1 efferocytosis signaling in muscle macrophages.

**Conclusions:**

Our data demonstrate that muscle inflammation can be suppressed potentially by activating the intrinsic TGF-β signaling in myofibers to promote IL-10 dependent-macrophages efferocytosis.

Video Abstract

**Supplementary Information:**

The online version contains supplementary material available at 10.1186/s12964-023-01163-8.

## Introduction

Efferocytosis, programmed clearance of apoptotic cells, is the critical arm of inflammatory regression [1, 2]. Through efferocytosis, injured tissue has a better tendency of inflammatory elimination, immune defense, repair and regeneration. Furthermore, dangerous tissue maintains the normal inner micro-environment and metabolism balance [3–5]. DNA fragmentation, apoptosis-associated protein release, and the caspase activation have been described in skeletal muscle as a consequence of muscle trauma, disuse, limb unloading, genetic defect involving dystrophy or age-related myopenia [6–9]. As well, apoptosis of myoblast, multinucleated myofibers, stromal cells, fibro-adipogenic precursors (FAPs), or inflammatory leukocytes have also been reported in inflammatory myositis [10–14] and in exercise-induced muscle damage [15–17]. Macrophages efferocytosis and the transition from M1 to M2 phenotype are causally related and represent the limiting steps for muscle repairing. Macrophages efferocytosis thus modulates the homeostatic response during skeletal muscle injury, inflammation and healing procedure. On the contrary, defects in macrophages efferocytosis process may have deleterious effects, lead maladaptive tissue repairing with fat and collagen accumulation, and even trigger muscle autoimmunity [14].

TGF-β signaling is needed for alternatively activating M2 macrophages exerting efferocytosis function. Macrophages lacking TGF-βr2 cannot express a set of genes forming M2 polarizing program hallmarks [18, 19]. In addition, macrophage is a critical producer of TGF-β signal, which is activated by the uptake of apoptotic cells in macrophages and elicits anti-inflammatory effect [20]. TGF-β signaling participates in the proliferation and differentiation of muscle tissue and the apoptosis of exudative lymphocytes, inhibiting the continuation of inflammatory exudation of muscle injury, and accelerating the healing and fibroatization of muscle injury. It is also considered to be a major initiator of extracellular matrix (ECM) deposition and tissue fibrosis [21, 22]. In damaged muscle, after engulfment of apoptotic cells and necrotic myofibers, macrophages secreted TGF-β signaling, which induced the transfer from the inflammatory M1 to anti-inflammatory M2 phenotype, prompting to resolve muscle inflammation [20, 23, 24]. In addition, TGF-β signaling has been reported to contribute to muscle repair and regeneration, by reducing muscle inflammatory response and control myocyte immune behaviors, through modulating of secretion of IL-6, HLA class I and adhesion molecules (e.g. LFA-1) in myocytes [25–27]. Whether and how TGF-β signaling regulates efferocytosis capacity of macrophage in inflamed muscle remains to be elucidated.

It is reported that suppressing muscle TGF-β signaling protects myofibers from degeneration and prompt to muscle regeneration after acute myoinjury [28, 29]. Our recent work demonstrated a persistent elevation of TGF-β signaling in the inflamed muscle area and in regenerating myofibers. Furthermore, we revealed that, for SM TGF-βr2^−/−^ mice, the aggravated muscle inflammation and increased macrophages accumulation was observed in inflamed muscle [30], which suggesting the endogenous TGF-β signaling of skeletal muscle devoted to the activation and function of macrophages. It still remains to be delineated whether myofiber-specific TGF-β signaling has a role in the development of local muscle inflammation after myoinjury, through regulating macrophages efferocytosis in inflamed muscle.

In this exploration, the role of the intrinsic TGF-β signaling of myofibers on macrophages infiltration, phenotypes, polarization and clearance for apoptotic cells in inflamed muscle in control or SM TGF-βr2^−/−^ mice after Cardiotoxin (CTX) injection was explored. Meanwhile, the details of the regulation of myofiber with TGF-β signaling activation on macrophages efferocytosis pathway molecules were addressed.

## Materials and methods

### Ethical approval

All animal experiments were approved by Animal Experimentation Ethics Committee of Southern Medical University (Approval No. L2016068).

### Mouse strains and animal experiments

C57BL/6 (B6) mice were provided by Animal Experimentation Centre of the Southern Medical University. Mice with TGF-βr2 knockout in skeletal muscle (designated as ‘SM TGF-βr2^−/−^mice’) were generated by crossing MCK-Cre mice (Jackson Lab) with floxed TGF-βr2 (TGF-βr2^flox/flox^, Jackson Lab). The genotypes of the generated mice was checked by polymerase chain reaction (PCR) with mouse tail DNA (Tab.1). TGF-βr2^flox/flox^ mice were used as the control for SM TGF-βr2^−/−^ mice.

**Table 1:** Primer sequences used for phenotype identification of SM TGF-βr2^−/−^ mice

Gene	Prime Sequences(5’-3’)
Cre	For: GTGAAACAGCATTGCATTGCTGTCACTT Rev: TAAGTCGAACCCGGTCTGCCAA
TGF-βr2^flox/flox^	For: TAAACAAGGTCCGGAGCCCA Rev: ACTTCTGCAAGAGGTCCCCT

Male mice of 6–8 weeks old were used. The total of 30 μL Cardiotoxin (CTX) solution (50 μg/mL; Sigma, USA) was injected into unilateral tibialis anterior (TA) muscles to prepare mice myoinjury model, after Buprenorphine (0.05 mg/kg) anaesthesia.The sham mice were dealt with PBS. Animals were socially housed with a 12 h light–dark cycle, and euthanized by cervical dislocation on day 4, 7 and 10 post-injury. For analysis of gene, protein and histology, TA muscle specimens were collected and snap frozen with liquid nitrogen-cooled isopentane. For evaluating the exogenous apoptotic cell uptake by macrophages in inflamed muscle, mononuclear cells were extracted from B6 mice spleen by density gradient centrifugation according to the instructions of Ficoll Plus 1.083 (Solarbio, China), rendered apoptotic by UV-irradiation (254 nm, 30 min) and labeled with PKH67 membrane-intercalating dye (1:250, Zeye, China). Apoptotic cells (1 × 10^6^) were injected into damaged TA muscle 12 h before muscle sample collection.

### Primary cell cultures and pro-inflammatory stimulus

Murine myogenic precursor cells (MPCs) were collected from the limb muscle of neonatal control or SM TGF-βr2^−/−^mice. After Collagenase II (Sigma, USA) digestion, muscles homogeneous slurry was filtered and centrifuged for preparing the single-cell suspension. Mice Satellite Cell Isolation Kit (Miltenyi Biotec, Germany) was used to isolate MPCs. In brief, the isolated cells were resuspended, treated with Enzyme A, incubated, and then added Satellite Cell Isolation solution. Cell suspension was applied onto the LS column, placed in the magnetic field of a suitable MACS Separator (Miltenyi Biotec, Germany), and collected flow-through containing unlabeled cells, representing the enriched satellite cells. The collected cells were cultured in DMEM/F12 (HyClone, USA), containing 10% fetal bovine serum (FBS, Gibco, USA) and penicillin–streptomycin (100 μg/mL, Heraeus, Germany). When the cultured MPCs cover 70–80% of the cell culture dish area, the growth medium was substituted by a differential medium (adding 2% horse serum) for 72 h to differentiate the cells into myotubes (MPC-Myotubes). For pro-inflammatory stimuli, MPC-Myotubes were treated with lipopolysaccharide (LPS, 100 ng/mL, R&D, USA) and IFN-γ (3 ng/mL, R&D, USA).

For collecting peritoneal macrophages, the complete thioglycollate medium (2 mL, Sigma, USA) were injected into B6 mice peritoneal cavity. The mice were euthanized at 72 h and macrophages were isolated from peritoneal lavage fluid. Macrophages were cultured in DMEM containing 10% FBS (Gibco, USA) and penicillin–streptomycin (100 μg/mL, Heraeus, Germany).

### In vitro cell co-culture model and efferocytosis analysis

After 72 h horse serum differentiation, control or SM TGF-βr2^−/−^ mice derived MPC-Myotubes received 24 h treating with LPS and IFN-γ. Then, MPC-Myotubes were co-cultured with peritoneal macrophages for 4 h at a ratio of 1:2 (MPC-Myotubes: macrophages). Next, PKH67-labelled apoptotic or non-apoptotic cells (3 × 10^6^ cells) were added into co-cultures 45 min prior to efferocytosis assays, respectively. For re-activating TGF-β/Smad signaling in TGF-βr2 deficient MPCs-Myotubes, myotubes were cultured with Smad agonist SRI-011381 hydrochloride (SRI, 8 μg/mL, MedChemExpress, USA) before co-culturing. For changing IL-10 level, rmIL-10 (50 ng/mL, R&D, USA), or IL-10 antagonist AS101 (300 nM, Santa Cruz, USA) were added to culture media during co-culture. The above chemical administration was about 4 h prior to efferocytosis assays.

### Transcriptome assay

Total RNA samples of the damaged TA muscle were prepared and performed the global transcriptal expression analysis using the mouse one color GE 4 × 44 K G4846A, V2 Microarray Kit (G4140-90,040, Agilent Technologies). Following by the manufacturer’s protocols, construction of chip array, sample preparation, biomolecular reaction and signal detection were performed.

For microarray experiments of the cultured MPC-Myotubes, RNA from SM TGF-βr2^−/−^ or control mice derived and 72 h-differentiated MPC-Myotubes was extracted and maintained at -80 °C until used. A “GeneChip Mouse Transcriptome Array 1.0” was used to determine gene expression profile by Affymetrix platform at the Thermo Fisher (USA) containing > 23,000 protein coding genes. Quality of total RNA from MPC-Myotubes was confirmed with NanoDrop 1000 (ThermoFisher, USA) and analyzed by qubit® 3.0 Fluorometer (ThermoFisher, USA). Following by Affymetrix recommended protocols, synthesis, labeling and hybridization were performed after RNA extraction from three independent cell samples of each condition.

### Quantitative real-time PCR analysis

Total RNA from the cultured MPC-Myotubes, muscle samples, or macrophages sorting by FACS, were extracted following by TRIzol reagent (Invitrogen, USA). And then, 1 µg RNA was reverse transcripted (RT) to cDNA following the relative kit instructions (Fermentas, USA). Adding specific primers, RT-qPCR was performed in triplicate with a fluorescence-labeled SYBR Green/ROX qPCR Master Mix kit (ThermoFisher, USA) by an ABI Step One Plus system (Applied Biosystems, USA). The relative mRNA levels of TGF-β2, TGF-βr2, Arg1, Retnla, iNOS, Mrc1, TGF-β, IL-4, IL-6, IL-10, normalized to glyceraldehyde-3-phosphate dehydrogenase (GAPDH), were detected (Tab.2). The ^△△^ Ct method was used to calculate the relative mRNA levels and 2-^△△^ Ct were presented for fold changes (arbitrary units).

**Table 2:** Primer sequences used for PCR

Gene	Prime Sequences(5’-3’)
TGF-β2	For: GGCGGTGCTCGCTTTGTA Rev: TCCCGAATGTCTGACGTATTGA
TGF-βr2	For: GTGAGACTGTCCACTTGCGA Rev: TGTCGTTCTTCCTCCACACG
Arg1	For: CTCCAAGCCAAAGTCCTTAGAG Rev: AGGAGCTGTCATTAGGGACA
Retlna	For: CCAATCCAGCTAACTATCCCTCC Rev: ACCCAGTAGCAGTCATCCCA
Mrc1	For: CTCTGTTCAGCTATTGGACGC Rev: TGGCACTCCCAAACATAATTTGA
iNOS	For: CTTCCGGGCAGCCTGTGAGACG Rev: ATCCCCAGGTGTTCCCCAGGTAGG

### Histological and immunofluorescence detection

Transverse frozen sections of snap-frozen TA muscle were made with a thickness of 6 µm. And then, either stained with hematoxylin & eosin (HE) or by immunofluorescence. For immunofluorescent detection in vitro, 4% paraformaldehyde (Aladdin, China) was used to fix cultured macropahges in co-cultured systems for 20 min. 0.1% Triton X-100 was used to permeabilize the macrophages above for 10 min before being washed twice in PBS. The following antibodies were labeled respectively: rabbit polyclonal to CX3CR1, rabbit anti-p-STAT3, rabbit monoclonal to Rac1-GTP, rabbit monoclonal to CD206, rabbit polyclonal to Bcl3, rabbit polyclonal to Vav1,rabbit anti-mouse TGF-βr2, rat anti-mouse CD11b or F4/80, rabbit polyclonal anti-Dystrophin, rabbit anti-mouse p-TGF-βr2, mouse anti-mouse p-Smad2/3, rabbit anti-mouse IL-10, mouse anti-Myosin Heavy Chain monoclonal antibody. The first antibodies above were purchased from ThermoFisher, Bioss, eBioscience, Merck Millipore or Abcam. All dilution ratios of the first antibodies above were 1:200. Anti-Tunel-FITC (5:50, Yeason, China). Goat anti-rabbit IgG-Alexa Fluor 488, goat anti-rat IgG-FITC, donkey anti-rabbit IgG-555, donkey anti-mouse IgG-555 or goat anti-rat IgG-Cy3 were labeled as the secondary antibodies, which were purchased from Santa Cruz in USA and their dilution ratios were 1:600. DAPI (Abcam, UK) was used to counterstained with Nuclei. Slides and cell climbing sheets were observed by an Olympus BX51 fluorescence microscope (Olympus, Japan). The positive cells number or the intensity of staining of slides and cell climbing sheets from three independent results, under 20 × magnification, was quantified by the Image-Pro-Plus software.

### Cell sorting and analysis of flow cytometry

Using 0.2% II type collagenase (Sigma, USA), inflamed TA muscles were collected and digested for 40 min at the condition of 37 °C. In vivo, the single cell suspension obtained from muscle homogenate was blocked. In vitro, cultured cells were digested with Trypsin (Sigma, USA), resuspended in ice cold PBS to obtain the single cell suspension. The following fluorescent antibodies were used: anti-CD45-Pacific Blue, anti-F4/80-PE, anti-CD11b-PE, anti-MHC-II-eFluor 450, anti-Ly6C-FITC, anti-CX3CR1-APC, anti-CD206-eFluor 700, anti-Bcl3-FITC, anti-CD31-APC, anti-IL-10-FITC, rabbit anti-p-STAT3-FITC, the antibodies above were purchased from ThermoFisher and their dilution ratios were 1:100; Other antibodies involved anti-Vav1-FITC (1:100, Biorbyt, USA), anti-Rac1-GTP-FITC (1:100, Proteintech, USA), anti-Tunel-FITC (5:50, Yeason, China), anti-Annexin-V-APC (5:100, Sigma, USA), anti-CRT-Alexa Fluor 647 (1:100, Abcam, UK), anti-PKH67-Alexa Fluor 647 (1:250, Zeye, China), anti-CD36-Alexa Fluor 700 (1:100, eBioscience, USA), and anti-PPARγ-FITC (1:100, Abcam, UK). To analyze the labeled cells, FACSAria II cell sorter with FlowJo software (BD Biosciences, USA) were used.

### Western blot analysis

The protein extraction kit (KeyGEN, China) was used to extract the cells or tissue proteins. The following protein antibodies were labeled: rabbit anti-mouse TGF-βr2 or TGF-β2 (1:500, eBioscience, USA), rabbit anti-mouse p-TGF-βr2 (1:200, Bioss, China), rabbit anti-mouse Smad2/3 (1:500, ThermoFisher, USA), mouse anti-mouse p-Smad2/3 (1:500, ThermoFisher, USA), mouse anti-mouse GAPDH (1:4000, ThermoFisher, USA). After being washed for three times, the membranes were incubated with the following secondary antibodies: HRP conjugated anti-rabbit IgG (1:5000, Fudebio, China) or HRP conjugated anti-mouse IgG (1:5000, CST, USA). The protein bands were exposured by the ECL detection system (Protein Simple, USA) and analyzed by ImageJ v1.42 software (National Institutes of Health, USA).

### Luminex analysis

After 12 h and 24 h culture, to obtain supernatants, cell culture medium was collected and centrifuged. Subsequently, the levels of special cytokines in supernatants, containing IFN-γ, Eotaxin, G-CSF, IL-1β, IL-1α, GM-CSF, IL-10, IL-3, IL-13, IL-6, MCP-1,IL-17A, RANTES, MIP-1α etc., were measured by Luminex xMap™ technology with Bio-Rad Bio-Lpex 200 apparatus (Bio-Rad Laboratoried, China).

### Statistical analysis

Statistical data Quantitative values were expressed as mean ± standard deviation (SD). One-way ANOVA Test was for multiple comparisons by using SPSS ver. 20.0 software (IBM, USA). *P* < 0.05 was performed as statistically significant.

## Results

### Up-regulation of myofiber specific TGF-β-Smad2/3 signaling affects the phenotype and efferocytosis capacity of macrophages in inflamed muscle

**Fig. 1:**
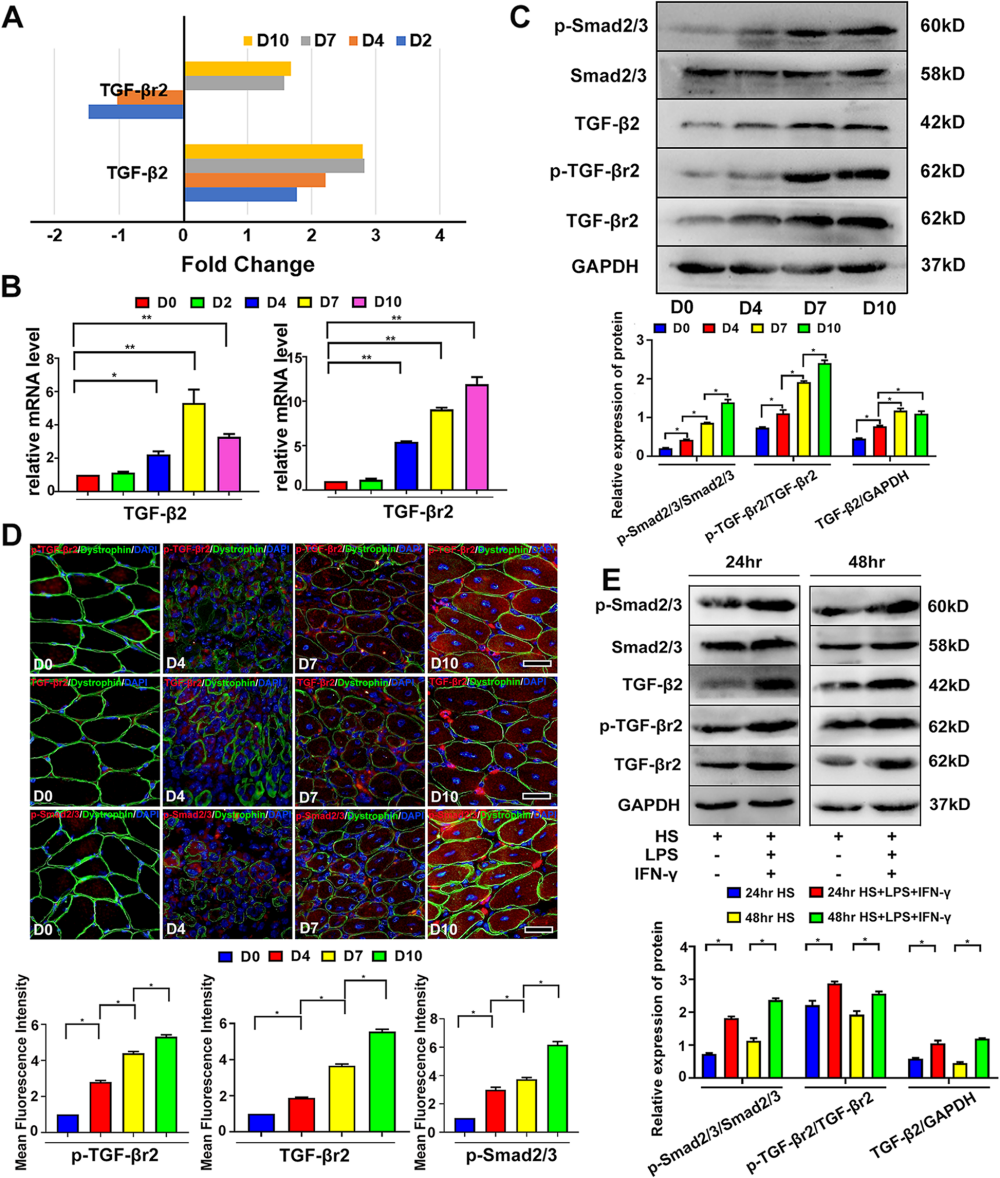
The inflammatory stimulus drives the myofiber specific TGF-β signal activation. **A**, RNA-seq analysis showing the mRNA level changes of TGF-β2 and TGF-βr2 of inflamed TA muscle. **B**, The mRNA levels of TGF-β2 and TGF-βr2 of inflamed muscle were quantified by qRT-PCR. **C**, Western blot analysis of protein levels in TGF-β2, p-TGF-βr2 and p-Smad2/3 in inflamed muscle. The relative protein level values were expressed as a ratio (protein of interest/GAPDH or phosphorylated (p) protein/total protein). **D**, Immunofluorescence double-staining results of TGF-βr2, p-TGF-βr2, p-Smad2/3 and Dystrophin in inflamed muscle. **E**, Western blot analysis of the protein levels of TGF-β2, p-TGF-βr2 and p-Smad2/3 in MPC-Myotubes receiving pro-inflammatory administration. Statistical data were expressed as the mean ± SD (*n* = 3). Multiple comparisons were analyzed by One-way ANOVA (*P < 0.05, **P < 0.01). Bar = 50 μm

As our microarray and PCR data shown, after CTX-myoinjury, gene levels of TGF-β2 and TGF-βr2 gradually up-regulated and reached the peak on days 7 and 10 in inflamed muscle of wild B6 mice (Fig. 1A,B). Tables 1 and 2. Also, our western blot analysis proved the up-regulation of TGF-β2, phosphorylated (p)-TGF-βr2 and phosphorylated (p)-Smad2/3 in inflamed muscle post-myoinjury (Fig. 1C). Using immunofluorescence staining, we further observed that, after CTX-myoinjury, the protein levels of TGF-βr2, p-TGF-βr2 and p-Smad2/3 elevated in the Dystrophin^+^ regenerating centronuclear myofibers (Fig. 1D). In our in vitro experiment, we also noticed the marked increase in protein expression of p-TGF-βr2 and p-Smad2/3 in murine MPC-Myotubes with pro-inflammatory administration (Fig. 1E). Since the first step of TGF-β signaling activation is TGF-β binding to TGF-βr2 [31], our data outlined that myofiber specific TGF-β signaling was driven to activation by the inflammatory stimuli.

**Fig. 2:**
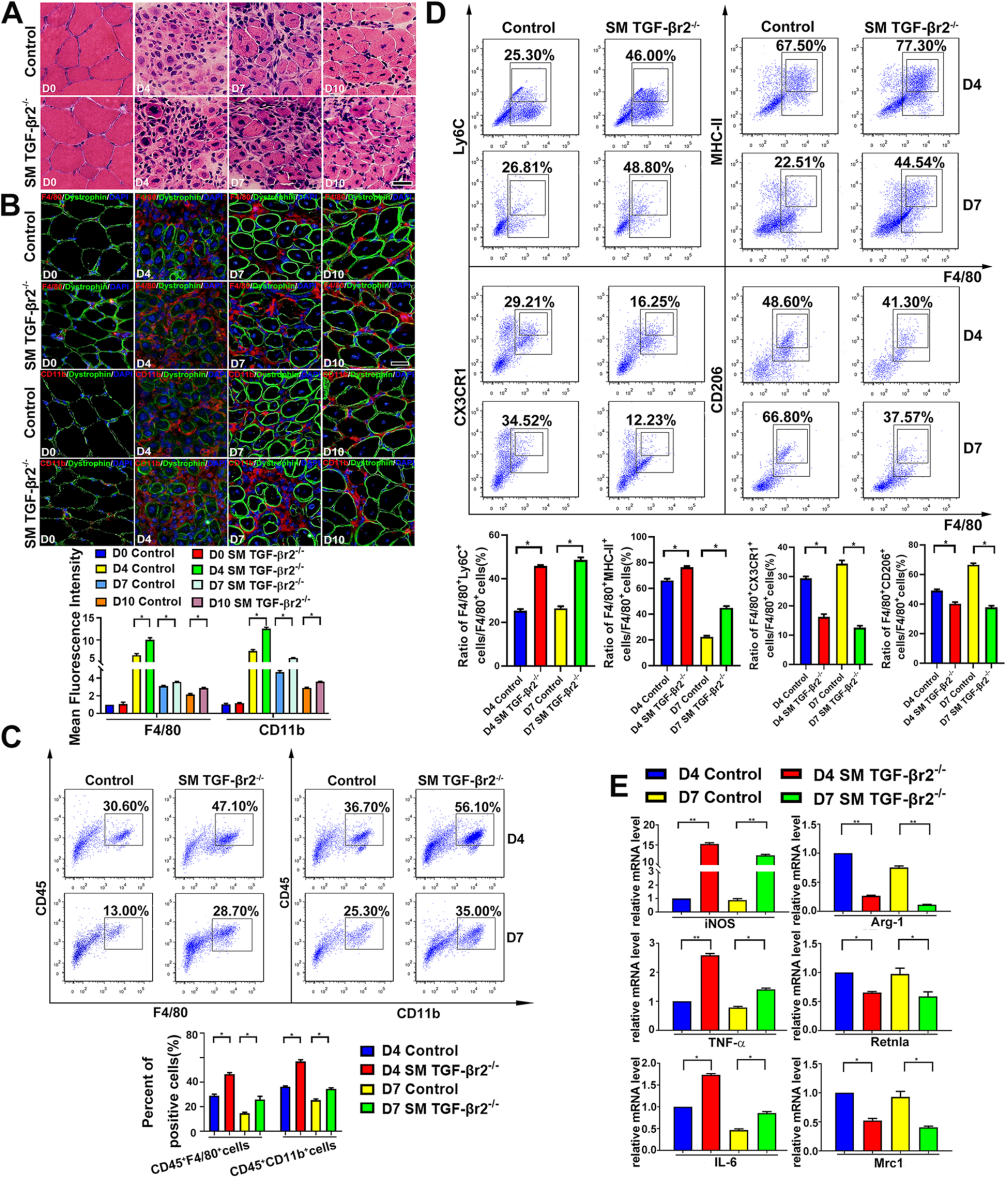
Myofiber TGF-β signaling deficiency exacerbated local muscle inflammation and impaired the transition of muscle macrophages from M1 to M2 phenotype in inflamed muscle. H&E staining (**A**) of CTX-damaged TA muscle. Immunofluorescence double-staining (**B**) of F4/80, CD11b and Dystrophin. FACS analysis of the proportion of CD45^+^CD11b^+^ **,** CD45^+^F4/80^+^ cells (**C**), M1 (F4/80^+^Ly6C^+^ or F4/80^+^MHC-II^+^) and M2 (F4/80^+^CD206^+^ or F4/80^+^CX3CR1^+^) cells (**D**). qRT-PCR analysis (**E**) in the mRNA levels of pro-inflammatory mediators (iNOS, TNF-α, IL-6) and anti-inflammatory mediators (Arg-1, Retlna, Mrc1) in sorted macrophages. Multiple comparisons were analyzed by One-way ANOVA. Statistical data were expressed as mean ± SD (*n* = 3). (*P < 0.05, **P < 0.01). Bar = 50 μm

For evaluating the role of myofiber specific TGF-β signaling on the muscle inflammatory response, we generated a transgenic model in which mice lack TGF-βr2 expression exclusively in skeletal muscle (SM TGF-βr2^−/−^ mice), through crossing TGFβr2-floxed mice with the MCK-Cre transgene mice (Fig.S1). The CTX myoinjury was performed in SM TGF-βr2^−/−^ and control mice respectively. Previous report indicated that specifically inhibiting TGF-β signaling in myofibers contributes to myorepair from acute injury [28]. In this work, we found myofiber TGF-β signaling deficiency aggravated muscle inflammatory reaction during the degeneration and the early myorepair stage after myoinjury (day 4, 7 and 10) (Fig. 2A), but similar to our recent report [30], eMHC re-expression in centronucleated myofibers showed no difference between control and SM TGF-βr2^−/−^ mice (Fig.S2). As shown through immunofluorescence and FACS analysis, muscle-specific TGF-β signaling deficiency led to the infiltration increase for CD45^+^CD11b^+^ and CD45^+^F4/80^+^ cells in inflamed muscle after myoinjury (Fig. 2B and C). Comparing to control mice, in inflamed muscle of SM TGF-βr2^−/−^ mice, the percent of pro-inflammatory M1 macrophages (F4/80^+^Ly6C^+^ or F4/80^+^MHC-II^+^) increased, but the percent of pro-resolving M2 cells (F4/80^+^CD206^+^ or F4/80^+^CX3CR1^+^) decreased post-myoinjury (day 4 and 7) (Fig. 2D). Our qPCR data further verified that, in macrophages sorted from inflamed muscle of SM TGF-βr2^−/−^ mice, gene levels of anti-inflammatory mediators, including Arg-1, Retlna and Mrc1 were significantly lower, but pro-inflammatory iNOS, IL-6 and TNF-α were significantly higher, comparing to control mice (Fig. 2E). Our data thus proved the myofiber TGF-β signaling deficiency exacerbated muscle inflammation and impaired the muscle macrophages transition from M1 to M2 phenotype.

**Fig.3:**
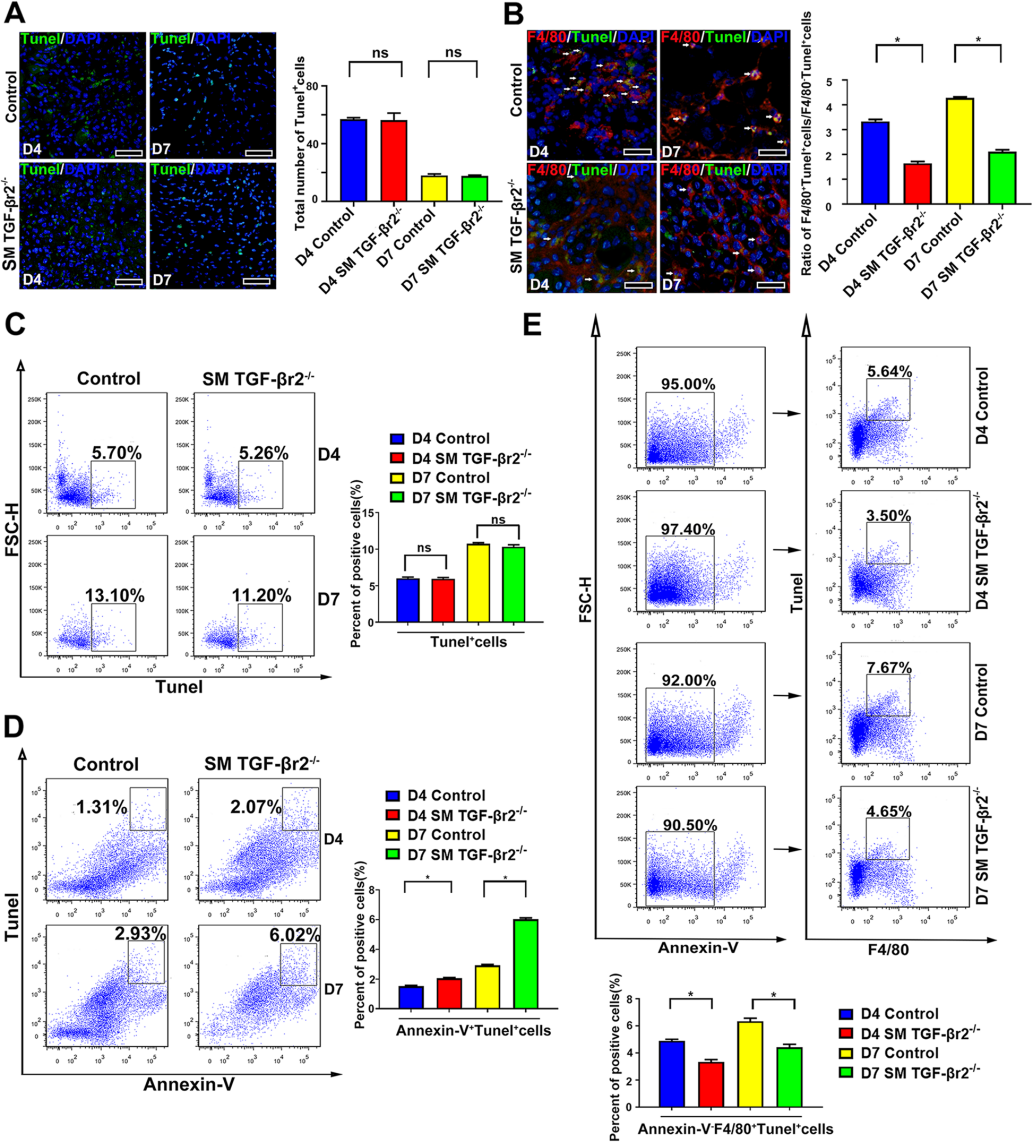
Myofiber specific TGF-β-Smad2/3 signaling effects on macrophages efferocytosis in inflamed muscle. Tunel staining of apoptotic cells (**A**). Immunofluorescence results of macrophages-associated apoptotic cells (F4/80^+^Tunel^+^) and free apoptotic cells (F4/80^−^Tunel^+^) (**B**). FACS analysis of the proportion of all apoptotic cells (Tunel^+^) (**C**), free apoptotic cells (Annexin-V^+^ Tunel^+^) (**D**) and efferocytotic macrophages (Annexin-V^−^F4/80^+^Tunel^+^) (**E**). Multiple comparisons were analyzed by One-way ANOVA. Statistical data were expressed as mean ± SD (*n* = 3). (*P < 0.05). Bar = 50 μm

In resolution stage after tissue injury, pro-resolving M2 macrophages mediate phagocytosis and clearance of apoptotic cells [1, 2]. We next questioned whether myofier specific TGF-β-Smad2/3 signaling might have effects on macrophages efferocytosis. Our immunofluorescence results showed that, on day 4 and 7 post-myoinjury, the total number of apoptotic cells (Tunel^+^) in inflamed muscles showed no significant difference between control and SM TGF-βr2^−/−^ mice (Fig. 3A), but in inflamed TGF-βr2^−/−^ muscle, the ratio of F4/80^+^Tunel^+^ cells to F4/80^−^Tunel^+^ cells (Efferocytotic marophages/ Free apoptotic cells) significantly reduced comparing to control muscle (Fig. 3B). A similar result was obtained by using FACS analysis. We found the absence of myofiber specific TGF-β signaling did not affect on the overall number of apoptotic cells (Tunel^+^) (Fig. 3C), but resulted in the increase of the percent of free apoptotic cells (Annexin-V^+^Tunel^+^) (Fig. 3D), and the decrease of the percent of efferocytosis macrophages (Annexin-V^−^F4/80^+^Tunel^+^) (Fig. 3E). We next investigated whether myofier TGF-β signaling is required for the protein expression of eat-me signal Calreticulin (CRT) in apoptotic cells or no-eat-me signal (CD31) in living cells sorted from inflamed muscle. Of note, no significant difference was found in the percent of Tunel^+^CRT^+^ cells or Annexin-V^−^CD31^+^ cells in damaged muscle between control and SM TGF-βr2^−/−^ mice (Fig.S3). These data suggest myofiber specific TGF-β signaling is required for efferocytosis signaling of macrophages, but not for recognition signaling of apoptotic cells.

### Myofiber mediates macrophages IL-10-Vav1-Rac1 efferocytosis signaling in inflamed muscle by priming intrinsic TGF-β signaling

**Fig.4:**
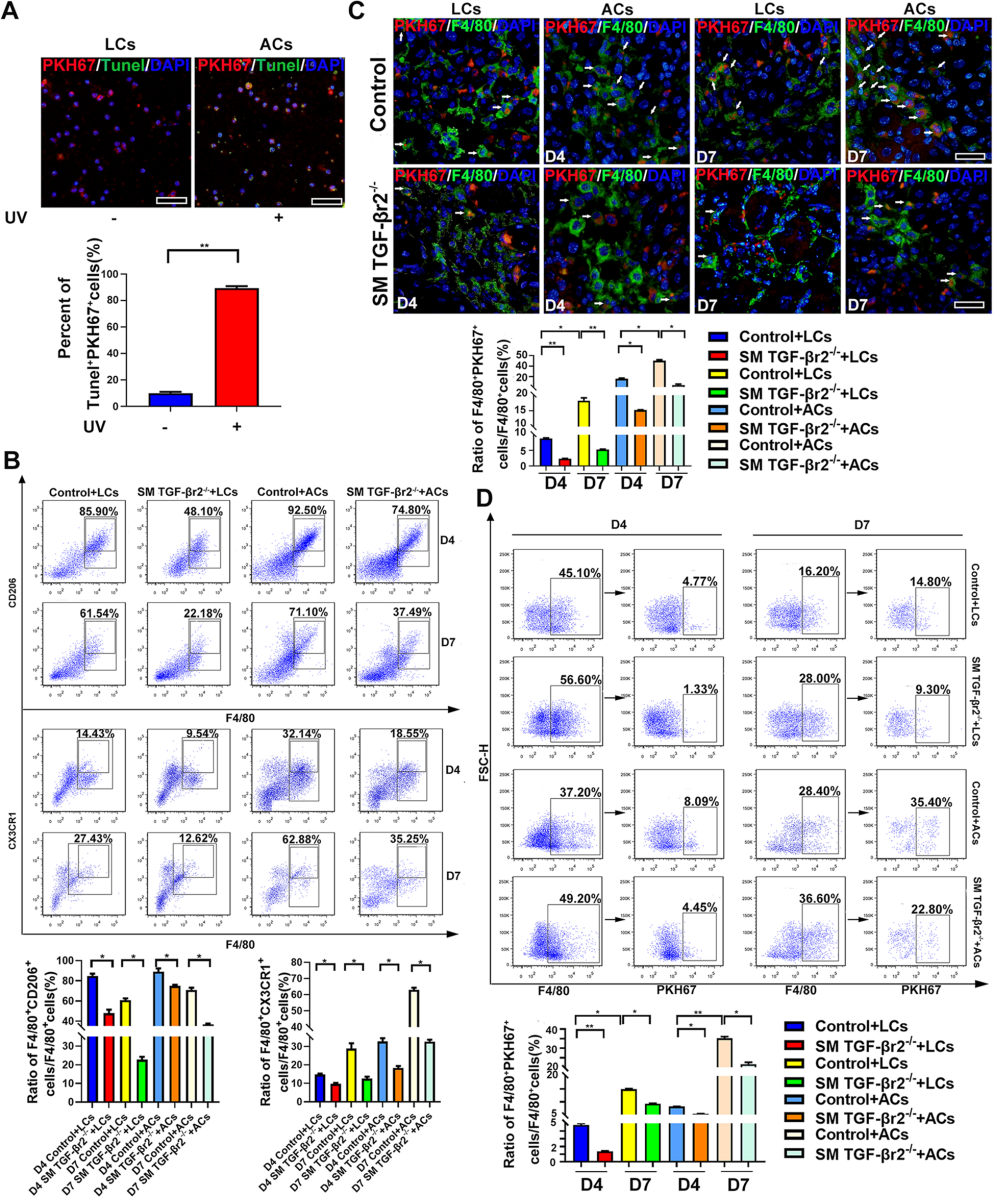
Myofiber drives macrophages efferocytosis by priming intrinsic TGF-β signaling. **A**, Immunofluorescence shows UV-inducted mononuclear cells apoptosis and PKH67 labeling. **B**, FACS analysis of the percent of M2 macrophages (F4/80^+^CD206^+^ or F4/80^+^CX3CR1^+^) sorted from inflamed muscle injected with exogenous ACs or not. **C**, Immunofluorescence shows the uptake of PKH67^+^ apoptotic cells in macrophages after exogenous apoptotic cells transferring into inflamed muscle. **D**, FACS analysis of the percent of efferocytosis macrophages (F4/80^+^PKH67^+^) sorted from inflamed muscle injected with exogenous ACs or not. LCs: Living cells; ACs: Apoptotic cells; Multiple comparisons were analyzed by One-way ANOVA. Statistical data were expressed as mean ± SD (*n* = 3). (*P < 0.05, **P < 0.01). Bar = 50 μm

As noted above, the intrinsic muscle TGF-β signaling has an effect on macrophages efferocytosis in inflamed muscle. For further validating this point, we extracted mononuclear cells from B6 mice spleen, rendered apoptotic by UV-irradiation and labeled with PKH67 membrane-intercalating dye (Fig. 4A). Apoptotic cells (ACs, PKH67^+^Tunel^+^) or living cells (LCs, PKH67^+^Tunel^−^) were then injected into damaged TA muscle 12 h before muscle sample collection. As shown through FACS, the exogenous ACs transfer resulted in a marked increase in the percent of M2 macrophages (F4/80^+^CD206^+^ or F4/80^+^CX3CR1^+^) in inflamed muscle of control mice (Fig. 4B). As well, ACs transfer into control mice enhanced the uptake of PKH67^+^ apoptotic cells in macrophages (Fig. 4C, D). Conversely, in damaged muscle of SM TGF-βr2^−/−^ mice, PKH67^+^ ACs uptake by macrophages was markedly impaired comparing to that in control mice, despite ACs transfer led to the increase of M2 and efferocytotic macrophages in inflamed TGF-βr2^−/−^ muscle (Fig. 4B-D). Our data thus confirmed myofier-driven macrophages efferocytosis by priming intrinsic TGF-β signaling.

**Fig.5:**
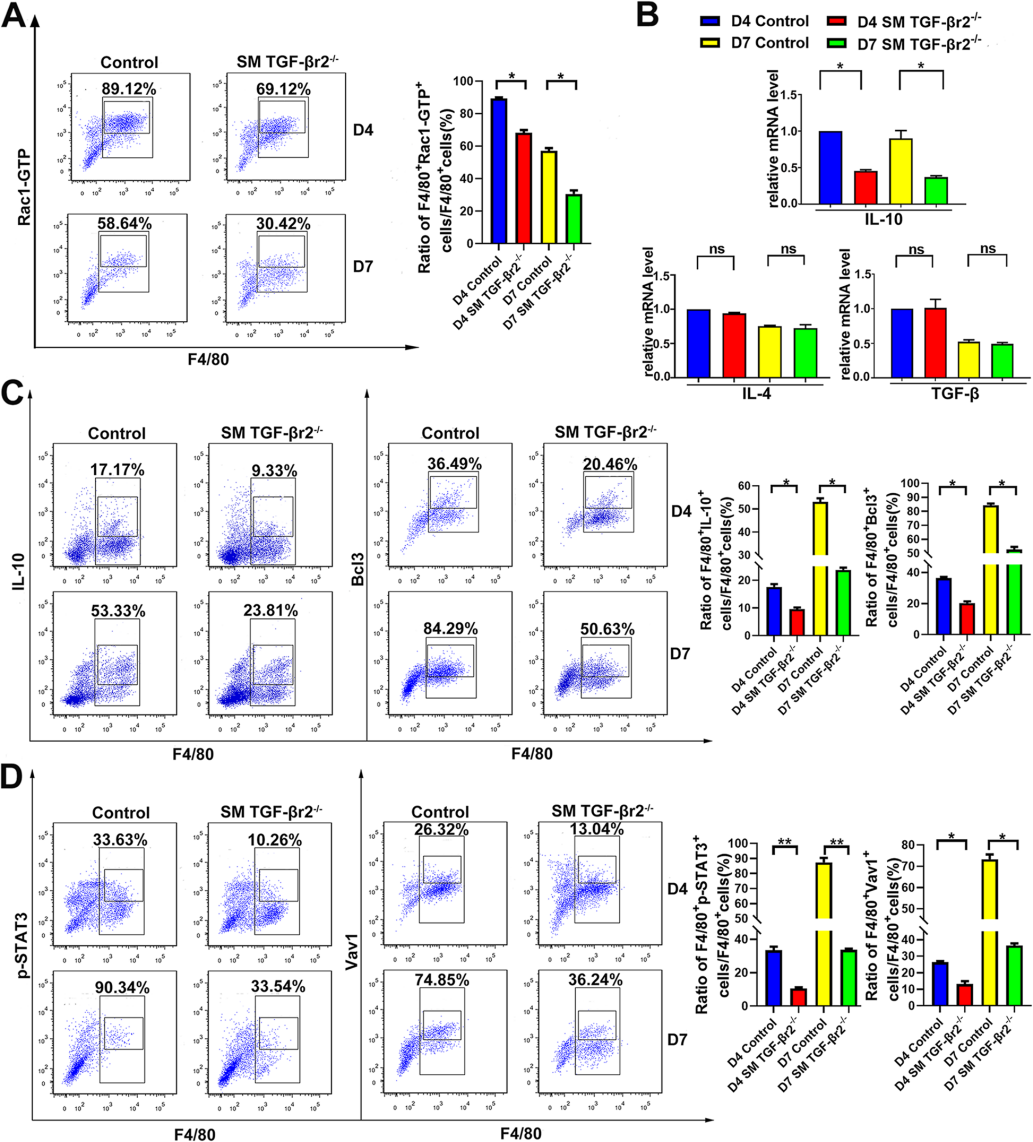
Myofiber TGF-β signaling mediates macrophages IL-10-Vav1-Rac1 efferocytosis signaling. FACS analysis of the expression of Rac1-GTP (**A**), IL-10 and Bcl3 (**C**), p-STAT3 and Vav1 (**D**) in macrophages sorted from inflamed muscle. mRNA levels of IL-10, IL-4 and TGF-β in sorted macrophages were analyzed by qRT-PCR (**B**). Multiple comparisons were analyzed by One-way ANOVA. Statistical data were expressed as mean ± SD (*n* = 3). (*P < 0.05, **P < 0.01)

Nuclear receptor PPARs is the key engulfment receptor regulator in macrophage [32, 33]. To uncover whether muscle specific TGF-β signaling is associated with PPARs pathway during macrophages efferocytosis, we analyzed M2-promoting nuclear receptor PPARγ and its downstream scavenger receptor CD36 in macrophages sorted from inflamed muscle of control and SM TGF-βr2^−/−^ mice. Anyway, PPARγ and CD36 expression in intramuscular macrophages showed no significant difference between two kinds of mice above (Fig.S4), suggesting the effect of myofiber TGF-β signaling on macrophages efferocytosis is independently from the nuclear engulfment receptor associated-efferocytosis signaling. Eat-me signaling in apoptotic cells stimulates the elevation of chemokine receptor CX3CR1 and efferocytotic receptors stabilin, αvβ3 and αvβ5 integrin in macrophages. In particular, after the recognition of apoptotic cell membrane receptors (e.g. Mertk), Rac1-GTP elevates early and is recruited to the phagocytic cup as well as promoting F-actin assembly [34, 35]. We next wonder whether CX3CR1 and GTPase Rac1 in macrophages might be affected by muscle TGF-β signaling deficiency. As expected, in inflamed muscle, we monitored the markedly reduced number of CX3CR1^+^ (Fig. 2D) and Rac1-GTP^+^ (Fig. 5A) macrophages in SM TGF-βr2^−/−^ mice, comparing to control mice. Through the action of GEFs, Rac1 cycles between an active form Rac1-GTP and an inactive form Rac1-GDP. GEF Vav1, a responsive target of STAT3, has participated in macrophages efferocytosis as well as being one of the members of IL-10 transcriptional programming [36, 37]. We reasoned that the myofiber TGF-β signaling might involve in Vav1-Rac1 efferocytosis pathway induced by IL-10 in intramuscular macrophages. As expected, our PCR data showed a lower gene level of IL-10 in muscle macrophages sorted from SM TGF-βr2^−/−^ mice, than from control mice (Fig. 5B). Through FACS analysis, we also monitored the decreased percent in macrophages expressing IL-10 and Bcl3 (target gene of IL-10) (Fig. 5C) and the reduced proportion of F4/80^+^p-STAT3^+^ and F4/80^+^Vav1^+^ cells sorted form SM TGF-βr2^−/−^ mice (Fig. 5D). Further, we found inhibiting TGF-β signaling in myofibers had no effects on gene levels of TGF-β and IL-4 in macrophages (Fig. 5B), which also involving in the activation of STAT3 or Vav1 in efferocytotic macrophages [38, 39]. Taken together, these data suggest an IL-10-Vav1-Rac1 efferocytosis signaling pathway in muscle macrophages, through a myofiber TGF-β signaling-dependent mechanism.

### Muscle-specific TGF-β signaling inhibition reduces macrophage efferocytic capacity in inflamed muscle by controlling IL-10 production

**Fig.6:**
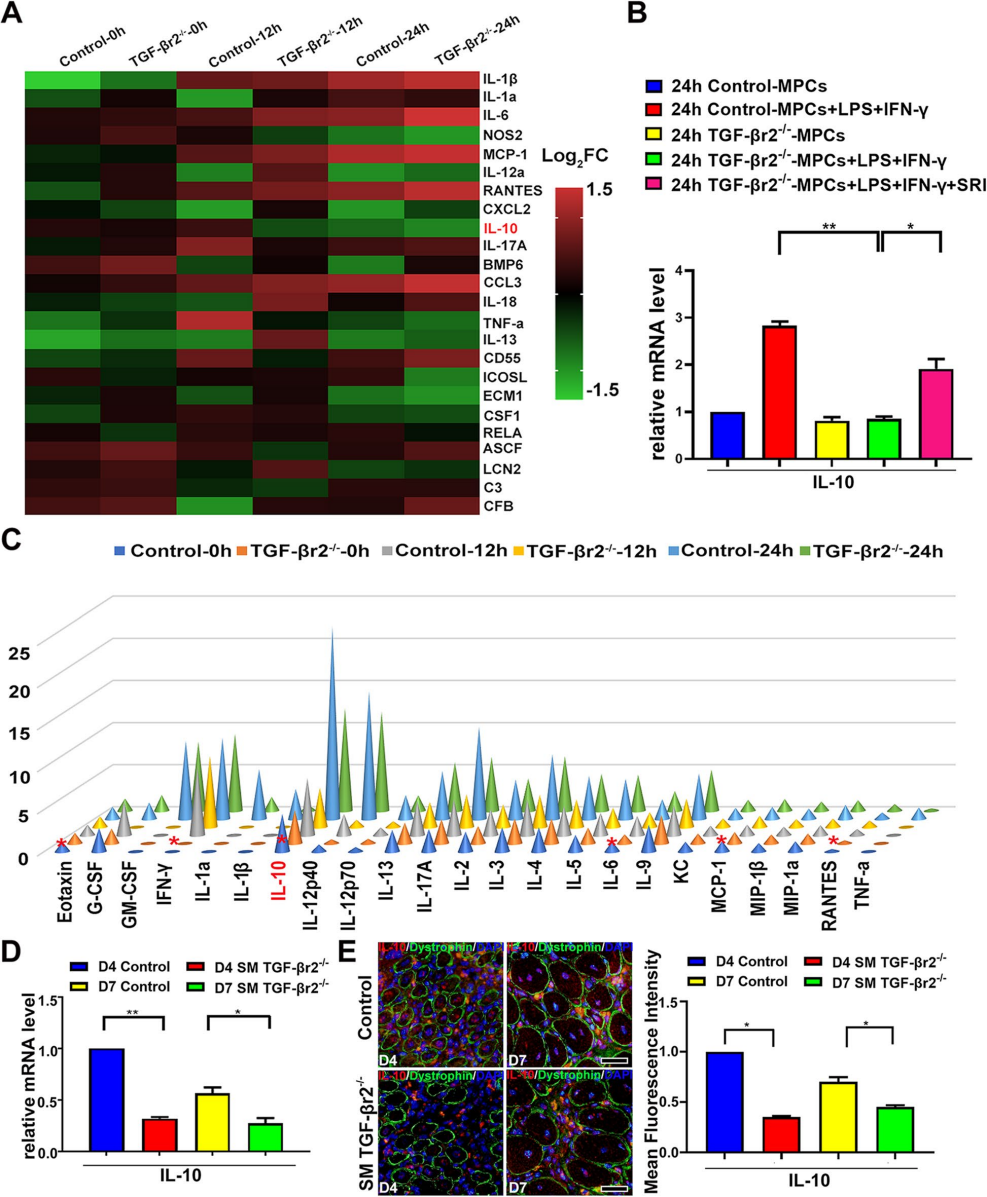
Endogenous TGF-β signaling regulates IL-10 production in regenerating myofiber. Transcriptome assay (**A**), qRT-PCR analysis (**B**) and Luminex analysis (**C**) of gene levels of some myokines, mRNA level of IL-10, protein levels of some myokines in TGF-βr2^−/−^- or control-MPC-Myotubes exposed to pro-inflammatory milieu, respectively. qRT-PCR analysis (**D**) of IL-10 mRNA level in inflamed muscle. Immunofluorescence staining (**E**) showed IL-10 expression change in regenerating myofibers. Multiple comparisons were analyzed by One-way ANOVA. Statistical data were expressed as mean ± SD (*n* = 3). (*P < 0.05, **P < 0.01). Bar = 50 μm

Next we furtherly explored how TGF-βr2^−/−^ myofiber modulates intramuscular macrophages efferocytosis. Muscle cells are capable of producing muscle-derived cytokines (myokines) in response to inflammatory stimuli and therefore can participate in the regulation of muscle inflammation [27]. Thus, we questioned whether myofibers from SM TGF-βr2^−/−^ mice might regulate the specific myokines linked with macrophages efferocytosis. By mRNA array analysis with the in vitro differentiated MPC-Myotubes, we demonstrated that inflammatory exposure induced the gene level elevation for some pro-inflammatory myokines in TGF-βr2^−/−^-MPC- Myotubes, involving IL-1, IL-6, RANTES, MCP-1, CCL3, etc. (Fig. 6A). However, we found IL-10 mRNA level in TGF-βr2^−/−^-MPC-Myotubes was significantly lower than that of control MPC-Myotubes (Fig. 6A, B). Of note, adding of Smad pathway agonist SRI corrected gene level of IL-10 in TGF-βr2^−/−^-MPC- Myotubes (Fig. 6B). Further, our Luminex analysis verified the decrease of IL-10 protein level in primary TGF-βr2^−/−^-MPC-Myotubes exposed to pro-inflammatory milieu (Fig. 6C). As expectedly, when we turned to in vivo test, we found on day 4 and 7 post-injury, IL-10 gene level prominently decreased in inflamed muscle (Fig. 6D), and IL-10 protein expression reduced in regenerating myofibers of SM TGF-βr2^−/−^ mice, comparing to control mice (Fig. 6E). Since IL-10 enhances efferocytosis by macrophages [40], we here infer that, TGF-βr2^−/−^ myofibers mediate macrophages efferocytosis through controlling on myokine IL-10 production.

**Fig.7:**
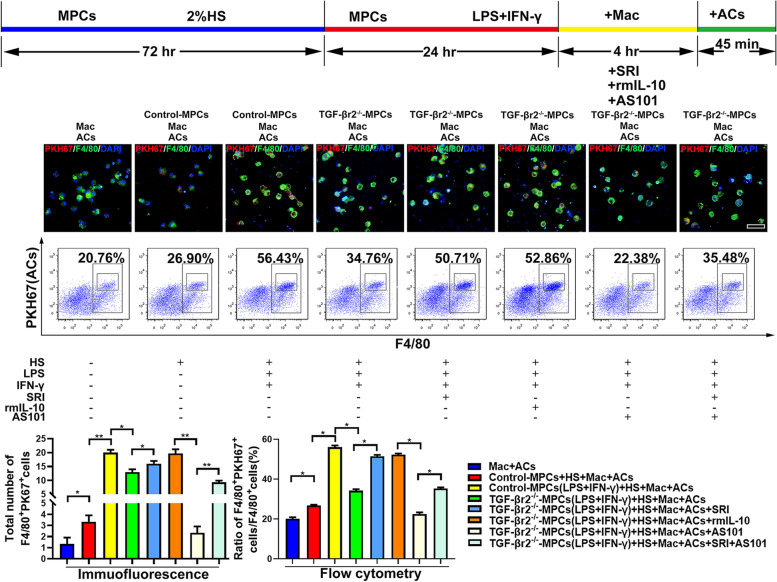
Myofiber TGF-β-IL-10 signaling effects on macrophages efferocytosis. Immunofluorescence staining (upper part) and FACS analysis (lower part) of the uptake of PKH67 labeled apoptotic cells by macrophage co-cultured with TGF-βr2^−/−^- or control-MPC-Myotubes exposed to pro-inflammatory milieu, and treated with or without SRI, rmIL-10 or AS101. Mac: Macrophage; ACs: Apoptotic cells; Multiple comparisons were analyzed by One-way ANOVA. Statistical data were expressed as mean ± SD (*n* = 3). (*P < 0.05, **P < 0.01). Bar = 50 μm

**Fig.8:**
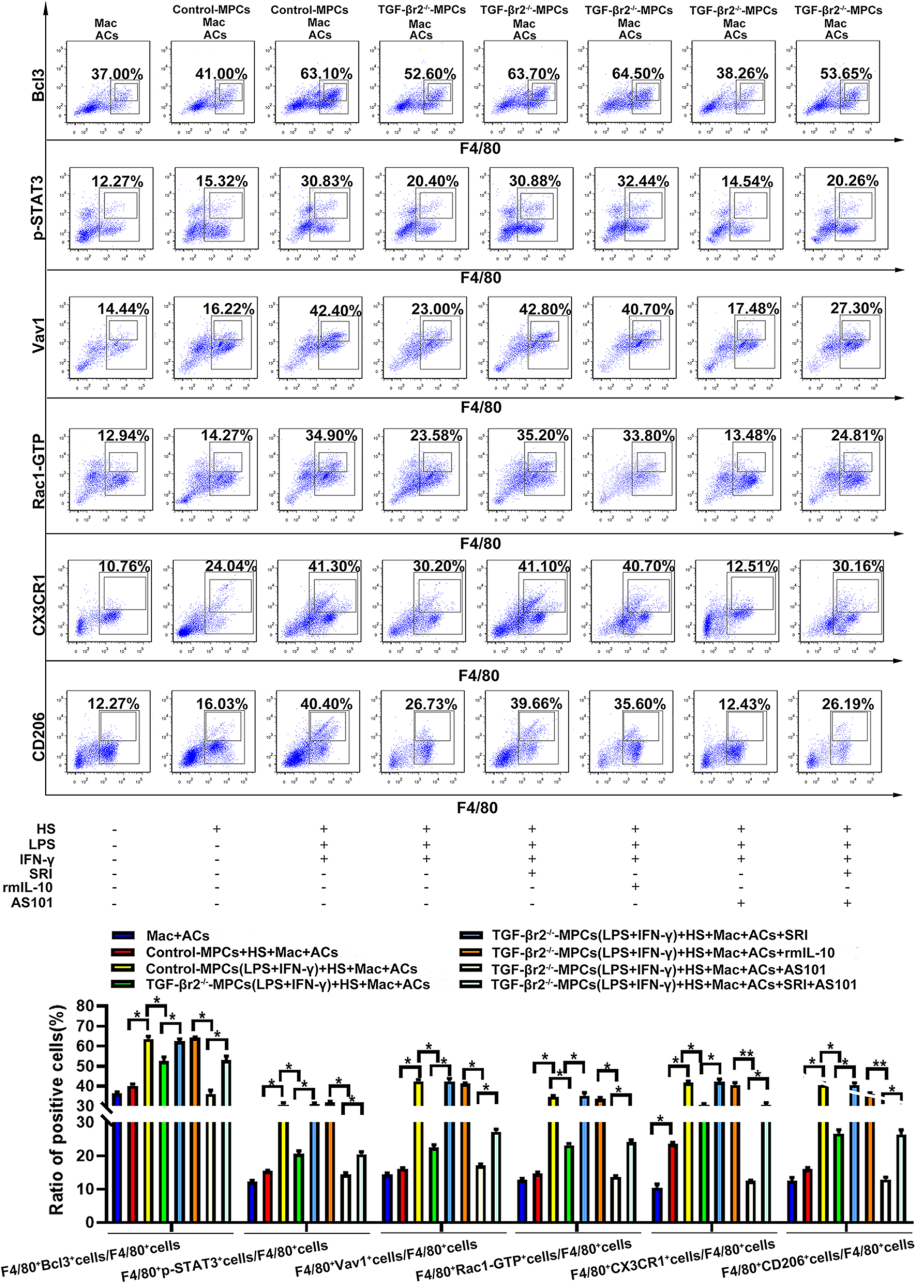
Myofiber TGF-β-IL-10 signaling effects on macrophage phenotype and efferocytosis related-molecules expression. FACS analysis of Bcl3, p-STAT3, Vav1, GTPase Rac1, CD206 and CX3CR1 in macrophages co-cultured with TGF-βr2.^−/−^- or control-MPC-Myotubes, exposed to pro-inflammatory milieu, and treated with or without SRI, rmIL-10 or AS101, respectively. Multiple comparisons were analyzed by One-way ANOVA. Statistical data were expressed as mean ± SD (*n* = 3). (*P < 0.05, **P < 0.01)

For definitively refvealing the suppressive effects of the myofiber TGF-β-IL-10 signaling on macrophages efferocytosis, we next turned to the cell co-culture model in vitro. For that, MPCs were collected from muscle of neonatal SM TGF-βr2^−/−^ or control mice and cultured in IFN-γ-LPS-containing pro-inflammatory milieu, with 2% horse serum (HS) for forming myotube. After 24 h, MPC-Myotubes were rinsed and co-cultured with the isolated macrophage from B6 mice abdominal cavity for 4 h, and then incubated with PKH67-labeled apoptotic cells (PKH67^+^ ACs) for 45 min. And then, we rinsed and removed the unengulfed ACs, and fixed the macrophages immediately. As predicted, our fluorescence staining and FACS analysis displayed that, macrophages enhanced PKH67^+^ ACs uptake in response to control-MPC-Myotubes (Fig. 7). Whereas, TGF-βr2^−/−^-MPC-Myotubes failed to boost macrophages efferocytosis, as presented by the lower percent of PKH67^+^F4/80^+^ cells in co-culture system (Fig. 7). Instead, the adding Smad agonist SRI, or recombinant IL-10 to the assays could markedly increase the percent of PKH67^+^ macrophages co-cultured with TGF-βr2^−/−^-MPC-Myotubes (Fig. 7). Otherwise, adding both SRI and IL-10 antagonist AS101 had no effect on the percent of PKH67-positivity in macrophages co-cultured with TGF-βr2^−/−^-MPC-Myotubes (Fig. 7). It is reported that, through an autocrine-paracrine pathway of IL-10, macrophages enhance the internalization of apoptotic cell by activating IL-10-mediated Rac1 signaling [41]. Therefore, our data imply that myofiber TGF-β signaling mediates IL-10-dependent macrophages efferocytosis by paracrine manner. To furtherly test this point, we detected efferocytosis related-molecules expression in macrophages co-cultured with TGF-βr2^−/−^- or control-MPC-Myotubes. We showed that, comparing to control-MPC-Myotubes, the adding of TGF-βr2^−/−^-MPC-Myotubes markedly down-regulated efferocytosis molecules expression in macrophages, involving Bcl3, p-STAT3, Vav1 and GTPase Rac1 (Fig. 8, S5). Consistently, TGF-βr2^−/−^ -MPC-Myotubes co-culture also inhibited the expression of the pro-resolving M2 molecule CD206 and CX3CR1 in macrophages incubated with ACs (Fig. 8, S5). Of note, the expression of efferocytosis and M2 molecules in macrophages could be corrected by the additional adding of SRI or recombinant IL-10 (Fig. 8, S5). Importantly also, TGF-βr2^−/−^-MPC-Myotubes induced-decrease of the above molecules in macrophages could not be rescued by adding of both SRI and AS101 (Fig. 8, S5). In summary, the above observations pointed toward an unknown effects of myofiber-intrinsic TGF-β signaling on Vav1-Rac1 efferocytosis signaling in intramuscular macrophages, by directing the production of IL-10 in regenerating myofibers after CTX myoinjury.

## Discussion

TGF-β signaling transduction is the basis of various cellular functions involving in proliferation, differentiation, motility and cell death. Cell types and the context in which the signal is received can determine the exact biologic response to TGF-β signal [42]. Despite the enhanced TGF-β activity is linked with intramuscular inflammatory response and myorepair procedure after myoinjury [43–45], it remains unclear whether and how the muscle specific-TGF-β signaling is relevant to intramuscular macrophage phagocytosis functions. Our results provide the first evidence that myofiber is a mediator of macrophages efferocytosis through its intrinsic TGF-β signaling activation following skeletal muscle damage.

In efferocytotic macrophages, post-engulfment signaling play important roles in controlling phagocytosis [32]. PPARγ, the member of nuclear receptor (NR) family, is a critical regulator of macrophages efferocytosis [46, 47]. According to our data, the role of myofiber TGF-β signaling on macrophages efferocytosis is independently from the nuclear engulfment receptor associated-efferocytosis signaling, since we monitored muscle TGF-β signaling deficiency had no effect on PPARγ and CD36 expression in intramuscular macrophage. Some cytokines have been reported to enhance efferocytosis capacity of macrophages. For example, as an efferocytosis-induced resolving/repair protein, TGF-β production elevated in macrophages undergoing efferocytosis [38]. In addition, IL-4 has been proven to transform macrophages from phenotype M1 to phenotype M2 in many diseases [39]. Through PCR and Luminex analysis, we exclude the effects of myofiber TGF-β signaling on macrophages efferocytosis by priming these two cytokines related-phagocytosis pathway. Rac subfamily GTPases, a key regulator of apoptotic cell engulfment, have been emphasized in the process of phagocytosis. Based on data in previous reports [34, 35], different signal cascades in macrophages converge to activate GTPase Rac1, leading to the reorganization of actin and the uptake of the apoptotic cells. Vav1, the PH domain containing Rac guanine nucleotide exchange factors (GEFs), could activate Rac GTPase to polymerize F-actin, and enhance apoptotic cells uptake. STAT3, the mediator of IL-10 transcriptional programming, was induced by IL-10 signal to prompt apoptotic cell uptake via inducing Vav1 [36, 37]. Our findings showing that, muscle TGF-β signaling deficiency leads to the expression decrease of Rac1-GTP, p-STAT-3, Vav1, IL-10 and Bcl3 in muscle macrophages after acute myoinjury. While, we monitored a lower gene level of IL-10 in macrophages sorted from damaged muscle of SM TGF-βr2^−/−^ mice. Our current data suggest that, myofibers enhance the IL-10-Vav1-Rac1 signaling-associated apoptotic cell internalization in macrophages through an intracellular TGF-β signaling pathway.

As an anti-inflammatory cytokine, interleukin 10 (IL-10) plays a critical role in the regulation of immune responses, which inhibits the release of pro-inflammatory factors in a variety of cell types [48]. Recent studies suggested that, macrophages also are target cells of IL-10 inhibitory effects [49, 50]. In inflamed skeletal muscle, IL-10 activates M2 macrophages, which releases cytokines to deactivate M1 macrophages. The expression of IL-10 by M2 macrophages appears to be significant for myogenesis in the context of restricting secondary muscle damage and promoting myogenic cell activation, differentiation, and fusion [23, 24, 51]. Indeed, IL-10 had been used as the systemic inflammation biomarker to assess the anti-inflammatory effect of the exercise in atherosclerosis, myositis, and inflammatory myopathies [52–54]. Previous works emphasize the closer interactions between myofibers and the immune system, and indicated that cytokines and chemokines produced by muscle cells could coordinately recruit leukocytes for immune responses in inflamed muscle [55]. Some researchers believe that muscle cells do not synthesize IL-10 even under stimulatory conditions. However, Weijian chen et al. [56] reported that, the hybrid myotubes of primary satellite cells derived from C2C12 cells and the patient presented the high levels of IL-10 secretion under electrical pulse stimulation condition. In addition, IFN-γ induced-IL-10 up-regulation in C2C12 myoblasts were also reported [57, 58]. In this study, we monitored pro-inflammatory stimuli induced the elevation of IL-10 production in mice primary MPC-Myotubes. Moreover, we found IL-10 mRNA and protein level in TGF-βr2^−/−^-MPC-Myotubes was significantly lower than that in control-MPC-Myotubes under pro-inflammatory milieu. Of note, adding of Smad pathway agonist SRI rescued inhibition effect of myofiber TGF-β signaling deficiency on IL-10 production. In different cells, such as in Treg cells, TGF-β induces IL-10 production had been reported [19]. Our work reveals that, in myofibers, TGF-β-Smad signaling is necessary for myokine IL-10 production. It is reported that, via a paracrine pathway, IL-10 affects Vav1 and the GTPase Rac1, thus prompts macrophages to internalize apoptotic cells [41]. Our work thus suggests a molecular mechanism of macrophages efferocytosis in inflamed muscle mediated by myofibers TGF-β-Smad-IL-10 pathway.

## Conclusions

In conclusion, our findings demonstrate that, the reduced IL-10 production in regenerating myofibers with TGF-β signaling deficiency results in down-regulating macrophages efferocytosis in inflamed muscle. This occurs through the regulation of IL-10-Vav1-Rac1 efferocytosis signaling pathway in muscle macrophages (Fig.S6). Our work thus suggests a plausible link among myofiber, IL-10, and macrophage efferocytosis, and we believe myofibers-associated IL-10 production devotes to strengthening macrophages efferocytosis under inflammatory environment.

## Supplementary Information

**Additional file 1:** Myofiber directs macrophages IL-10-Vav1-Rac1 efferocytosis pathway in inflamed muscle following CTX myoinjury by arousing intrinsic TGF-β signaling. **Fig.S1.** Construction and identification of SM TGF-βr2^−/−^mice. Genomic PCR identification of SM TGF-βr2^−/−^ mice was showed. MCK-Cre^+^/TGF-βr2^flox/wt^: the lanes 1, 4, 7, 8, 10; MCK-Cre^+^/TGF-βr2^flox/flox^ : the lanes 3, 5, 9; MCK-Cre^+^/TGF-βr2^wt/wt^: the lanes 2, 6; Western blots analysis of TGF-βr2 protein expression in myocardium, gastrocnemius, tibialis anterior muscle and immunofluorescence staining resultsof TGF-βr2 protein expression in tibialis anterior muscle between controlandSM TGF-βr2^−/−^ mice. Statistical data were expressed as the mean± SD. Multiple comparisons were analyzed by One-way ANOVA. Bar = 50 μm. **Fig.S2**. Immunofluorescence double-staining results of eMHC and Dystrophin in inflamed TA muscle from SM TGF-βr2^-/-^ and control mice. The mean fluorescence intensity of eMHC was quantified. Statistical data were expressed as the mean±SD. Multiple comparisons were analyzed by One-way ANOVA. Bar = 50 μm. **Fig.S3**. Myofiber specific TGF-β signaling has no effect on the recognition signaling of apoptotic cells. FACS analysis of the expression of eat-me signal in apoptotic cellsand no-eat-me signal in living cellsin inflamed muscle from control and SM TGF-βr2^-/-^ mice. Multiple comparisons were analyzed by One-way ANOVA. Statistical data were expressed as mean±SD. **Fig.S4**. Myofiber TGF-β signaling shows no effect on the nuclear engulfment receptor associated-efferocytosis signaling in macrophage. FACS analysis of the expression of PPARγ and CD36 in macrophage sorted from inflamed muscle, between SM TGF-βr2^-/-^ and control mice. Multiple comparisons were analyzed by One-way ANOVA. Statistical data were expressed as mean±SD. **Fig.S5**. Immunofluorescence staining demonstrates the effects of myofiber TGF-β-IL-10 signaling on M2 phenotypeand efferocytosis molecules expressionin macrophages co-cultured with TGF-βr2^−/−^- or control-MPC-Myotubes, exposed to pro-inflammatory milieu, treated with or without SRI, rmIL-10 or AS101, respectively. Multiple comparisons were analyzed by One-way ANOVA. Statistical data were expressed as mean±SD.. Bar = 50 μm. **Fig.S6**. A proposed model depicting the mechanism by which myofibers modulating IL-10-Vav1-Rac1 macrophages efferocytosis signaling pathway in inflamed muscle through activating the intrinsic TGF-β signaling.

## Data Availability

Not applicable.
