# Supplementary figures and images for: Myofiber directs macrophages IL-10-Vav1-Rac1 efferocytosis pathway in inflamed muscle following CTX myoinjury by activating the intrinsic TGF-β signaling
Source: Cell Commun Signal. 2023 Jul 4;21:168. doi: 10.1186/s12964-023-01163-8 (PMC10318691; doi:10.1186/s12964-023-01163-8)

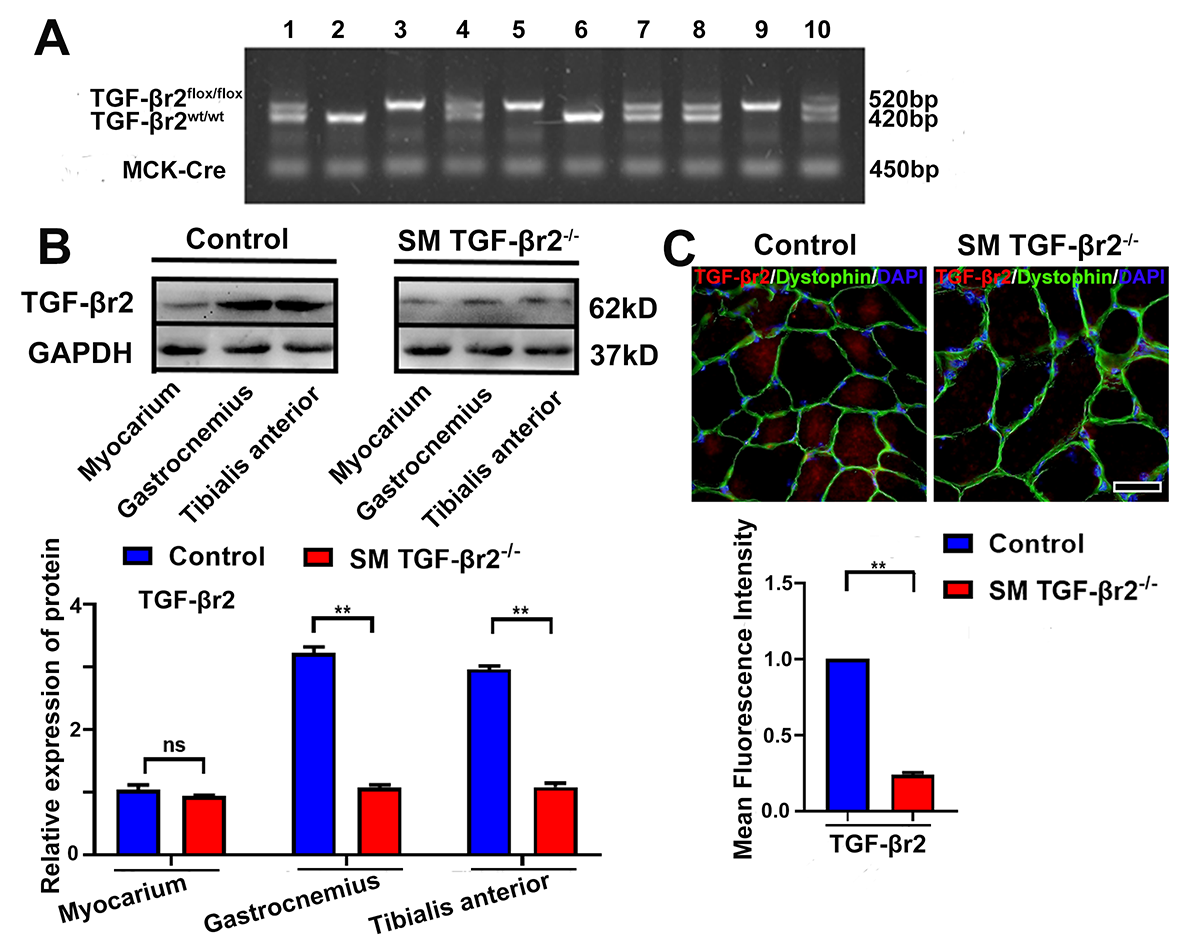

Supplement: Supplementary file 2 — Additional file 1: Myofiber directs macrophages IL-10-Vav1-Rac1 efferocytosis pathway in inflamed muscle following CTX myoinjury by arousing intrinsic TGF-β signaling. Fig.S1. Construction and identification of SM TGF-βr2−/−mice. Genomic PCR identification of SM TGF-βr2−/− mice was showed. MCK-Cre+/TGF-βr2flox/wt: the lanes 1, 4, 7, 8, 10; MCK-Cre+/TGF-βr2flox/flox : the lanes 3, 5, 9; MCK-Cre+/TGF-βr2wt/wt: the lanes 2, 6; Western blots analysis of TGF-βr2 protein expression in myocardium, gastrocnemius, tibialis anterior muscle and immunofluorescence staining resultsof TGF-βr2 protein expression in tibialis anterior muscle between controlandSM TGF-βr2−/− mice. Statistical data were expressed as the mean± SD. Multiple comparisons were analyzed by One-way ANOVA. Bar = 50 μm. Fig.S2. Immunofluorescence double-staining results of eMHC and Dystrophin in inflamed TA muscle from SM TGF-βr2-/- and control mice. The mean fluorescence intensity of eMHC was quantified. Statistical data were expressed as the mean±SD. Multiple comparisons were analyzed by One-way ANOVA. Bar = 50 μm. Fig.S3. Myofiber specific TGF-β signaling has no effect on the recognition signaling of apoptotic cells. FACS analysis of the expression of eat-me signal in apoptotic cellsand no-eat-me signal in living cellsin inflamed muscle from control and SM TGF-βr2-/- mice. Multiple comparisons were analyzed by One-way ANOVA. Statistical data were expressed as mean±SD. Fig.S4. Myofiber TGF-β signaling shows no effect on the nuclear engulfment receptor associated-efferocytosis signaling in macrophage. FACS analysis of the expression of PPARγ and CD36 in macrophage sorted from inflamed muscle, between SM TGF-βr2-/- and control mice. Multiple comparisons were analyzed by One-way ANOVA. Statistical data were expressed as mean±SD. Fig.S5. Immunofluorescence staining demonstrates the effects of myofiber TGF-β-IL-10 signaling on M2 phenotypeand efferocytosis molecules expressionin macrophages co-cultured with TGF-βr [file 12964_2023_1163_MOESM1_ESM.zip › Revised Supplementary files/FIG.S1(1Column).tif]

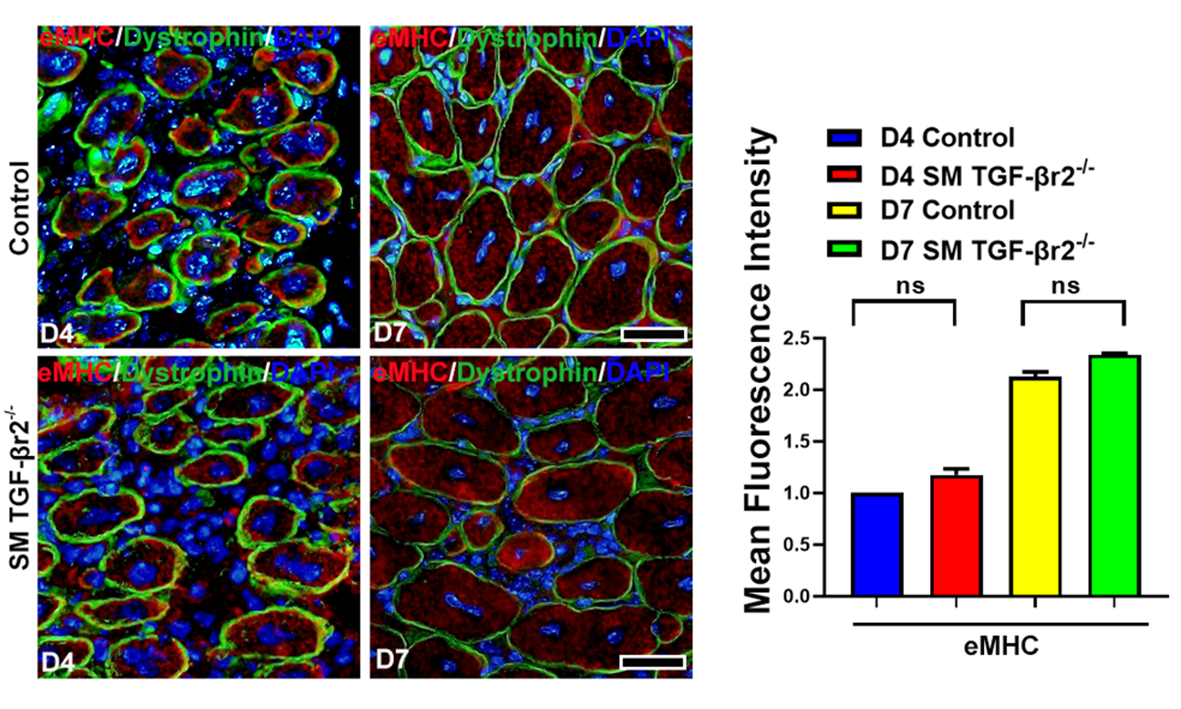

Supplement: Supplementary file 2 — Additional file 1: Myofiber directs macrophages IL-10-Vav1-Rac1 efferocytosis pathway in inflamed muscle following CTX myoinjury by arousing intrinsic TGF-β signaling. Fig.S1. Construction and identification of SM TGF-βr2−/−mice. Genomic PCR identification of SM TGF-βr2−/− mice was showed. MCK-Cre+/TGF-βr2flox/wt: the lanes 1, 4, 7, 8, 10; MCK-Cre+/TGF-βr2flox/flox : the lanes 3, 5, 9; MCK-Cre+/TGF-βr2wt/wt: the lanes 2, 6; Western blots analysis of TGF-βr2 protein expression in myocardium, gastrocnemius, tibialis anterior muscle and immunofluorescence staining resultsof TGF-βr2 protein expression in tibialis anterior muscle between controlandSM TGF-βr2−/− mice. Statistical data were expressed as the mean± SD. Multiple comparisons were analyzed by One-way ANOVA. Bar = 50 μm. Fig.S2. Immunofluorescence double-staining results of eMHC and Dystrophin in inflamed TA muscle from SM TGF-βr2-/- and control mice. The mean fluorescence intensity of eMHC was quantified. Statistical data were expressed as the mean±SD. Multiple comparisons were analyzed by One-way ANOVA. Bar = 50 μm. Fig.S3. Myofiber specific TGF-β signaling has no effect on the recognition signaling of apoptotic cells. FACS analysis of the expression of eat-me signal in apoptotic cellsand no-eat-me signal in living cellsin inflamed muscle from control and SM TGF-βr2-/- mice. Multiple comparisons were analyzed by One-way ANOVA. Statistical data were expressed as mean±SD. Fig.S4. Myofiber TGF-β signaling shows no effect on the nuclear engulfment receptor associated-efferocytosis signaling in macrophage. FACS analysis of the expression of PPARγ and CD36 in macrophage sorted from inflamed muscle, between SM TGF-βr2-/- and control mice. Multiple comparisons were analyzed by One-way ANOVA. Statistical data were expressed as mean±SD. Fig.S5. Immunofluorescence staining demonstrates the effects of myofiber TGF-β-IL-10 signaling on M2 phenotypeand efferocytosis molecules expressionin macrophages co-cultured with TGF-βr [file 12964_2023_1163_MOESM1_ESM.zip › Revised Supplementary files/FIG.S2(1Column).tif]

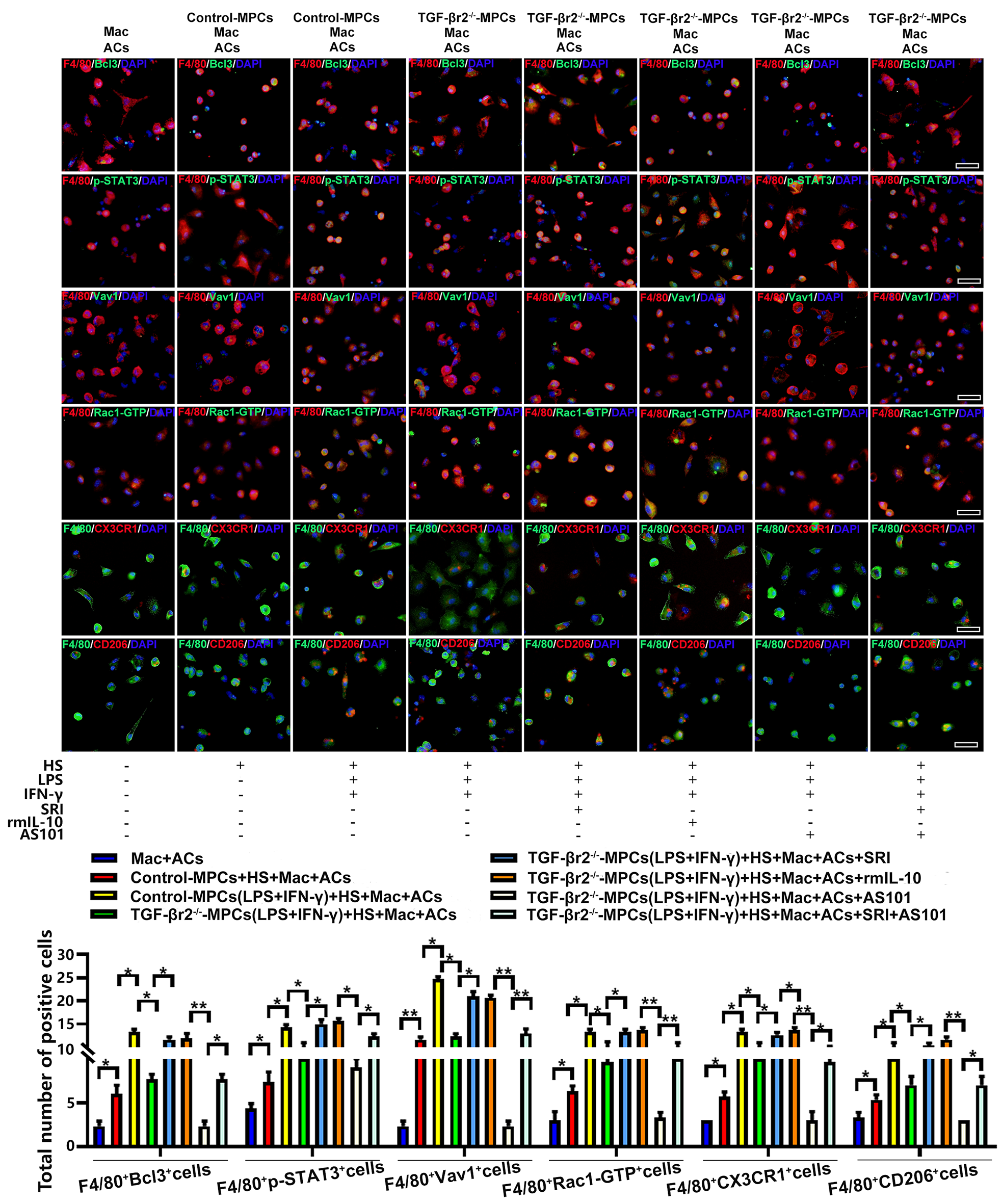

Supplement: Supplementary file 2 — Additional file 1: Myofiber directs macrophages IL-10-Vav1-Rac1 efferocytosis pathway in inflamed muscle following CTX myoinjury by arousing intrinsic TGF-β signaling. Fig.S1. Construction and identification of SM TGF-βr2−/−mice. Genomic PCR identification of SM TGF-βr2−/− mice was showed. MCK-Cre+/TGF-βr2flox/wt: the lanes 1, 4, 7, 8, 10; MCK-Cre+/TGF-βr2flox/flox : the lanes 3, 5, 9; MCK-Cre+/TGF-βr2wt/wt: the lanes 2, 6; Western blots analysis of TGF-βr2 protein expression in myocardium, gastrocnemius, tibialis anterior muscle and immunofluorescence staining resultsof TGF-βr2 protein expression in tibialis anterior muscle between controlandSM TGF-βr2−/− mice. Statistical data were expressed as the mean± SD. Multiple comparisons were analyzed by One-way ANOVA. Bar = 50 μm. Fig.S2. Immunofluorescence double-staining results of eMHC and Dystrophin in inflamed TA muscle from SM TGF-βr2-/- and control mice. The mean fluorescence intensity of eMHC was quantified. Statistical data were expressed as the mean±SD. Multiple comparisons were analyzed by One-way ANOVA. Bar = 50 μm. Fig.S3. Myofiber specific TGF-β signaling has no effect on the recognition signaling of apoptotic cells. FACS analysis of the expression of eat-me signal in apoptotic cellsand no-eat-me signal in living cellsin inflamed muscle from control and SM TGF-βr2-/- mice. Multiple comparisons were analyzed by One-way ANOVA. Statistical data were expressed as mean±SD. Fig.S4. Myofiber TGF-β signaling shows no effect on the nuclear engulfment receptor associated-efferocytosis signaling in macrophage. FACS analysis of the expression of PPARγ and CD36 in macrophage sorted from inflamed muscle, between SM TGF-βr2-/- and control mice. Multiple comparisons were analyzed by One-way ANOVA. Statistical data were expressed as mean±SD. Fig.S5. Immunofluorescence staining demonstrates the effects of myofiber TGF-β-IL-10 signaling on M2 phenotypeand efferocytosis molecules expressionin macrophages co-cultured with TGF-βr [file 12964_2023_1163_MOESM1_ESM.zip › Revised Supplementary files/FIG.S5(1.5 Column).tif]

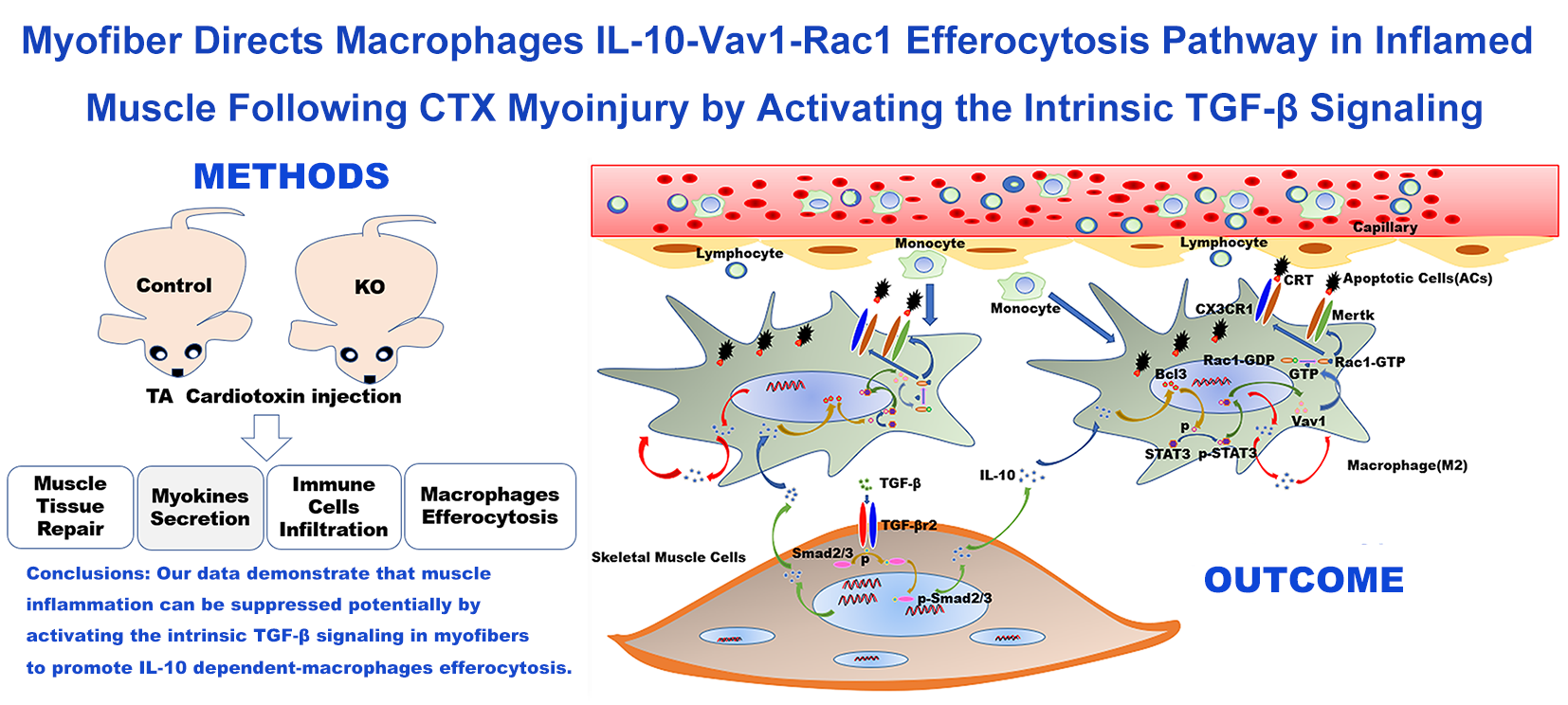

Supplement: Supplementary file 2 — Additional file 1: Myofiber directs macrophages IL-10-Vav1-Rac1 efferocytosis pathway in inflamed muscle following CTX myoinjury by arousing intrinsic TGF-β signaling. Fig.S1. Construction and identification of SM TGF-βr2−/−mice. Genomic PCR identification of SM TGF-βr2−/− mice was showed. MCK-Cre+/TGF-βr2flox/wt: the lanes 1, 4, 7, 8, 10; MCK-Cre+/TGF-βr2flox/flox : the lanes 3, 5, 9; MCK-Cre+/TGF-βr2wt/wt: the lanes 2, 6; Western blots analysis of TGF-βr2 protein expression in myocardium, gastrocnemius, tibialis anterior muscle and immunofluorescence staining resultsof TGF-βr2 protein expression in tibialis anterior muscle between controlandSM TGF-βr2−/− mice. Statistical data were expressed as the mean± SD. Multiple comparisons were analyzed by One-way ANOVA. Bar = 50 μm. Fig.S2. Immunofluorescence double-staining results of eMHC and Dystrophin in inflamed TA muscle from SM TGF-βr2-/- and control mice. The mean fluorescence intensity of eMHC was quantified. Statistical data were expressed as the mean±SD. Multiple comparisons were analyzed by One-way ANOVA. Bar = 50 μm. Fig.S3. Myofiber specific TGF-β signaling has no effect on the recognition signaling of apoptotic cells. FACS analysis of the expression of eat-me signal in apoptotic cellsand no-eat-me signal in living cellsin inflamed muscle from control and SM TGF-βr2-/- mice. Multiple comparisons were analyzed by One-way ANOVA. Statistical data were expressed as mean±SD. Fig.S4. Myofiber TGF-β signaling shows no effect on the nuclear engulfment receptor associated-efferocytosis signaling in macrophage. FACS analysis of the expression of PPARγ and CD36 in macrophage sorted from inflamed muscle, between SM TGF-βr2-/- and control mice. Multiple comparisons were analyzed by One-way ANOVA. Statistical data were expressed as mean±SD. Fig.S5. Immunofluorescence staining demonstrates the effects of myofiber TGF-β-IL-10 signaling on M2 phenotypeand efferocytosis molecules expressionin macrophages co-cultured with TGF-βr [file 12964_2023_1163_MOESM1_ESM.zip › Revised Supplementary files/FIG.S6(1.5Column).tif]

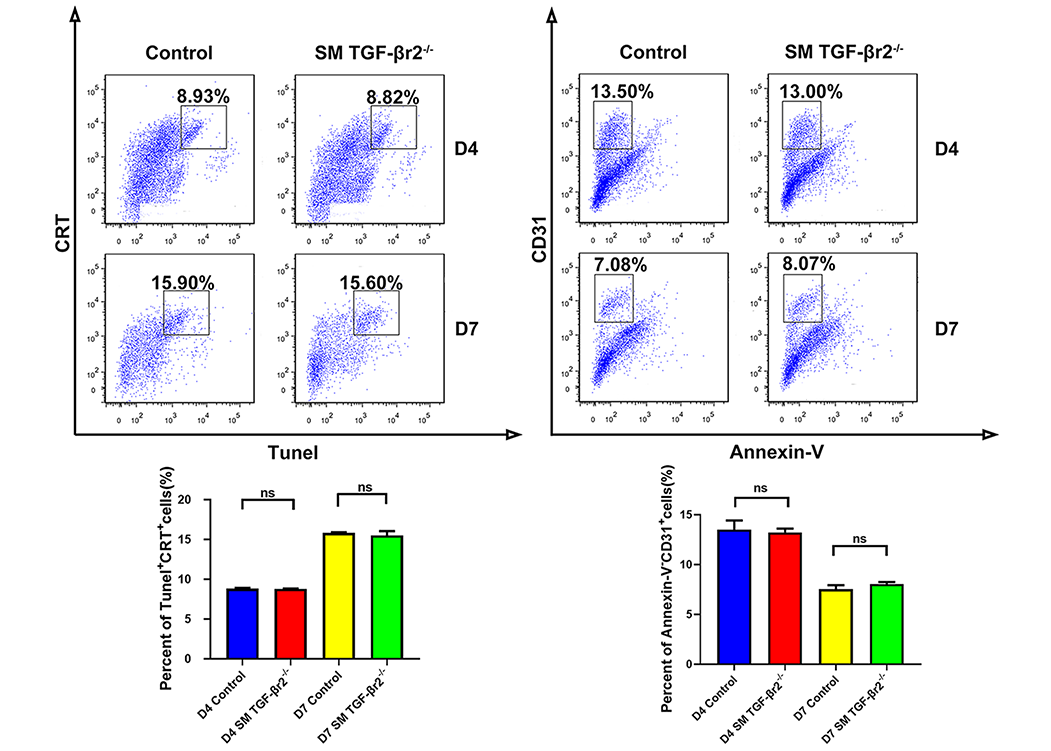

Supplement: Supplementary file 2 — Additional file 1: Myofiber directs macrophages IL-10-Vav1-Rac1 efferocytosis pathway in inflamed muscle following CTX myoinjury by arousing intrinsic TGF-β signaling. Fig.S1. Construction and identification of SM TGF-βr2−/−mice. Genomic PCR identification of SM TGF-βr2−/− mice was showed. MCK-Cre+/TGF-βr2flox/wt: the lanes 1, 4, 7, 8, 10; MCK-Cre+/TGF-βr2flox/flox : the lanes 3, 5, 9; MCK-Cre+/TGF-βr2wt/wt: the lanes 2, 6; Western blots analysis of TGF-βr2 protein expression in myocardium, gastrocnemius, tibialis anterior muscle and immunofluorescence staining resultsof TGF-βr2 protein expression in tibialis anterior muscle between controlandSM TGF-βr2−/− mice. Statistical data were expressed as the mean± SD. Multiple comparisons were analyzed by One-way ANOVA. Bar = 50 μm. Fig.S2. Immunofluorescence double-staining results of eMHC and Dystrophin in inflamed TA muscle from SM TGF-βr2-/- and control mice. The mean fluorescence intensity of eMHC was quantified. Statistical data were expressed as the mean±SD. Multiple comparisons were analyzed by One-way ANOVA. Bar = 50 μm. Fig.S3. Myofiber specific TGF-β signaling has no effect on the recognition signaling of apoptotic cells. FACS analysis of the expression of eat-me signal in apoptotic cellsand no-eat-me signal in living cellsin inflamed muscle from control and SM TGF-βr2-/- mice. Multiple comparisons were analyzed by One-way ANOVA. Statistical data were expressed as mean±SD. Fig.S4. Myofiber TGF-β signaling shows no effect on the nuclear engulfment receptor associated-efferocytosis signaling in macrophage. FACS analysis of the expression of PPARγ and CD36 in macrophage sorted from inflamed muscle, between SM TGF-βr2-/- and control mice. Multiple comparisons were analyzed by One-way ANOVA. Statistical data were expressed as mean±SD. Fig.S5. Immunofluorescence staining demonstrates the effects of myofiber TGF-β-IL-10 signaling on M2 phenotypeand efferocytosis molecules expressionin macrophages co-cultured with TGF-βr [file 12964_2023_1163_MOESM1_ESM.zip › Revised Supplementary files/Fig.S3(1 Column).tif]

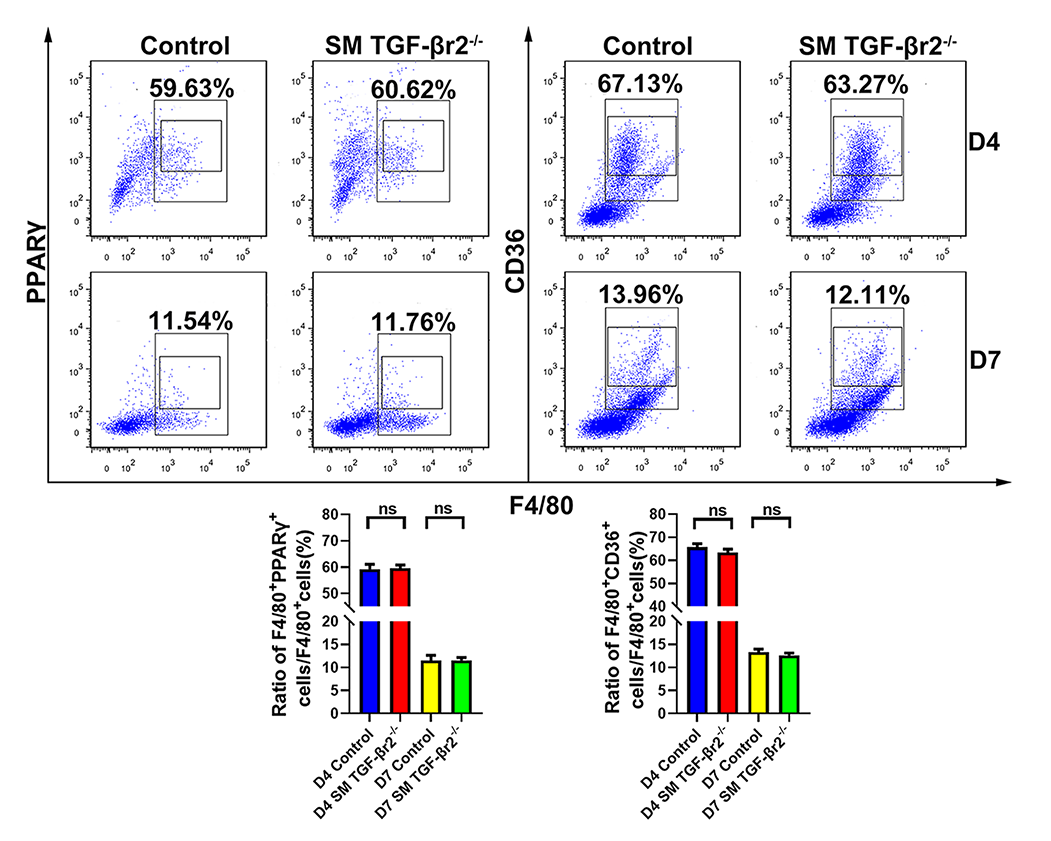

Supplement: Supplementary file 2 — Additional file 1: Myofiber directs macrophages IL-10-Vav1-Rac1 efferocytosis pathway in inflamed muscle following CTX myoinjury by arousing intrinsic TGF-β signaling. Fig.S1. Construction and identification of SM TGF-βr2−/−mice. Genomic PCR identification of SM TGF-βr2−/− mice was showed. MCK-Cre+/TGF-βr2flox/wt: the lanes 1, 4, 7, 8, 10; MCK-Cre+/TGF-βr2flox/flox : the lanes 3, 5, 9; MCK-Cre+/TGF-βr2wt/wt: the lanes 2, 6; Western blots analysis of TGF-βr2 protein expression in myocardium, gastrocnemius, tibialis anterior muscle and immunofluorescence staining resultsof TGF-βr2 protein expression in tibialis anterior muscle between controlandSM TGF-βr2−/− mice. Statistical data were expressed as the mean± SD. Multiple comparisons were analyzed by One-way ANOVA. Bar = 50 μm. Fig.S2. Immunofluorescence double-staining results of eMHC and Dystrophin in inflamed TA muscle from SM TGF-βr2-/- and control mice. The mean fluorescence intensity of eMHC was quantified. Statistical data were expressed as the mean±SD. Multiple comparisons were analyzed by One-way ANOVA. Bar = 50 μm. Fig.S3. Myofiber specific TGF-β signaling has no effect on the recognition signaling of apoptotic cells. FACS analysis of the expression of eat-me signal in apoptotic cellsand no-eat-me signal in living cellsin inflamed muscle from control and SM TGF-βr2-/- mice. Multiple comparisons were analyzed by One-way ANOVA. Statistical data were expressed as mean±SD. Fig.S4. Myofiber TGF-β signaling shows no effect on the nuclear engulfment receptor associated-efferocytosis signaling in macrophage. FACS analysis of the expression of PPARγ and CD36 in macrophage sorted from inflamed muscle, between SM TGF-βr2-/- and control mice. Multiple comparisons were analyzed by One-way ANOVA. Statistical data were expressed as mean±SD. Fig.S5. Immunofluorescence staining demonstrates the effects of myofiber TGF-β-IL-10 signaling on M2 phenotypeand efferocytosis molecules expressionin macrophages co-cultured with TGF-βr [file 12964_2023_1163_MOESM1_ESM.zip › Revised Supplementary files/Fig.S4(1 Column).tif]
